# Supplementary material for: A Novel Cartesian Plot Analysis for Fixed Monolayers That Relates Cell Phenotype to Transfer of Contents between Fibroblasts and Cancer Cells by Cell-Projection Pumping
Source: Int J Mol Sci. 2022 Jul 19;23(14):7949. doi: 10.3390/ijms23147949 (PMC9316567; doi:10.3390/ijms23147949)
Supplement: Supplementary file 1 [file ijms-23-07949-s001.zip › Supplementary Materials.pdf]

Supplementary Figures, Tables and MATLAB Script

for

A Novel Cartesian Plot Analysis for Fixed Monolayers That Relates Cell Phenotype to Transfer of Contents Between Fibroblasts and Cancer Cells by Cell-Projection Pumping

Swarna Mahadevan [1], Kenelm Kwong [1], Mingjie Lu [1], Elizabeth Kelly [1], Belal Chami [1,2], Yevgeniy Romin [3], Sho Fujisawa [3], Katia Manova [3], Malcolm A. S. Moore [4] and Hans Zoellner [1,4,5,6,7,*]

1 The Cellular and Molecular Pathology Research Unit, Oral Pathology and Oral Medicine, School of Dentistry, Faculty of Medicine and Health, The University of Sydney, Westmead Hospital, Westmead, NSW 2145, Australia; smah9946@uni.sydney.edu.au (S.M.); kenelm.kwong@health.nsw.gov.au (K.K.); mingjielu@gmail.com (M.L.); elizabeth.kelly@sydney.edu.au (E.K.); belal.chami@sydney.edu.au (B.C.)
2 The School of Medical Sciences, The University of Sydney, Sydney, NSW 2006, Australia;
3 Molecular Cytology, The Memorial Sloan Kettering Cancer Center, 415-417 E 68 Street, ZRC 1962, New York, NY, USA; rominy@mskcc.org (Y.R.); sfujisawa@hibercell.com (S.F.); k-manova@ski.mskcc.org (K.M.)
4 Cell Biology, The Memorial Sloan Kettering Cancer Center, 430 E 67th St, RRL 717, New York, NY 10065, USA; moorem@mskcc.org
5 Biomedical Engineering, Faculty of Engineering, The University of Sydney, Sydney, NSW 2006, Australia
6 Graduate School of Biomedical Engineering, University of NSW, Kensington, NSW 2052, Australia
7 Strongarch Pty Ltd., Pennant Hills, NSW 2120, Australia
* Correspondence: hans.zoellner@strongarch.com; Tel.: +61-466400028

Pages

2 to 14 Supplementary Figures

15 to 34 Supplementary Tables

35 to 80 MATLAB Script for Cartesian Plot Analysis

Supplementary Figures

Page	Figure
3	**Figure S1.** Graphical output of MATLAB script for Cartesian plot analysis of co-cultures of fibroblasts with SAOS-2 osteosarcoma cells in experiments 'a','b', 'c' and 'd'.
4	**Figure S2.** Graphical output of MATLAB script for Cartesian plot analysis of co-cultures of fibroblasts with SAOS-2 osteosarcoma cells in experiments 'e', 'f', 'g' and 'h'.
5	**Figure S3.** Graphical output of MATLAB script for Cartesian plot analysis of co-cultures of fibroblasts with osteosarcoma cell lines SAOS-2 in experiment 'i', and osteosarcoma U2OS cells in experiments 'a' and 'c'.
6	**Figure S4.** Graphical output of MATLAB script for Cartesian plot analysis of co-cultures of fibroblasts with MM200-B12 melanoma cells in experiments 'a', 'b', 'c' and 'e'.
7	**Figure S5.** Graphical output of MATLAB script for Cartesian plot analysis of co-cultures of fibroblasts with MeIRMu, and WM175 melanoma cells in experiments 'a' and 'c'.
8	**Figure S6.** Graphical output of MATLAB script for Cartesian plot analysis of a co-culture of human gingival fibroblasts WM175 melanoma cells in experiment 'c'.
9	**Figure S7.** Graphical output of MATLAB script for Cartesian plot analysis of co-cultures of fibroblasts with Colo316 colon carcinoma cells in experiments 'a', 'b', 'c' and 'd'.
10	**Figure S8.** Graphical output of MATLAB script for Cartesian plot analysis of co-culture of DiO pre-labelled human gingival fibroblasts with DiD pre-labelled PEO1 ovarian cancer cells, in experiment 'c'.
11	**Figure S9.** FACS plots of cancer cell lines (Colo316, SAOS-2, U2OS, MM200-B12, NM39, WM175) and human dermal fibroblasts pre-labelled with both possible dye orientations of DiD and DiO in experiments 'k' and 'l'..
12	**Figure S10.** Photomicrographs from experiment 'p' of control SAOS-2 pre-labelled with DiO and cultured in isolation for 24h, and then further labelled with propidium iodide for DNA and by fluorescence immunohistochemistry for 5mc.
13	**Figure S11.** Photomicrographs from experiment 'p' of control human dermal fibroblasts pre-labelled with DiD and cultured in isolation for 24h, and then further labelled with propidium iodide for DNA and by fluorescence immunohistochemistry for 5mc.
14	**Figure S12.** Cartesian plot analysis results for experiment 'n' for cell circularity and cell profile area.

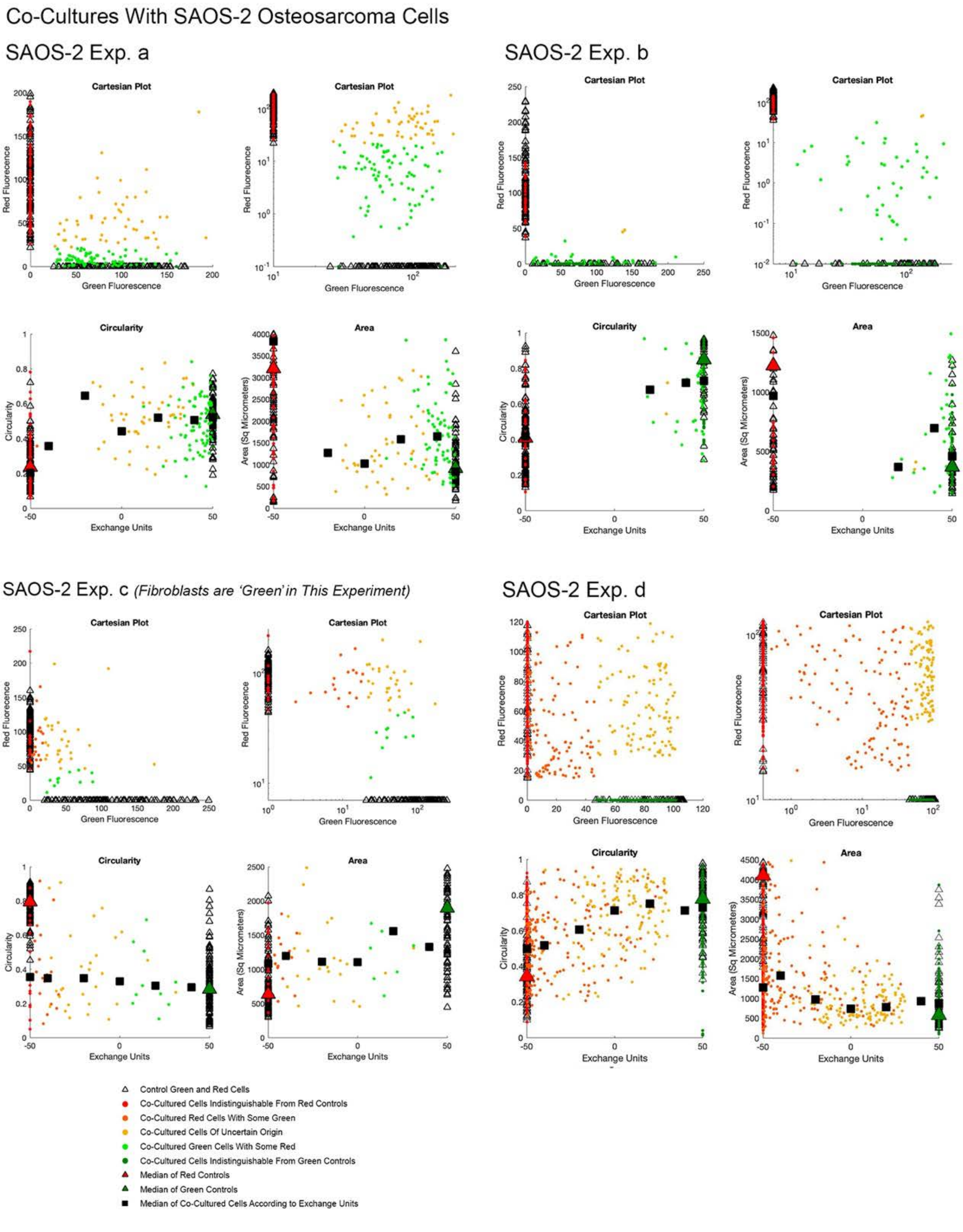

Figure S1. Graphical output of MATLAB script for Cartesian plot analysis of co-cultures of fibroblasts with SAOS-2 osteosarcoma cells in experiments 'a','b', 'c' and 'd'. Human dermal fibroblasts pre-labelled with DiD were in experiments 'a', 'b' and 'd', and SAOS-2 were pre-labelled with DiO in those experiments. Human gingival fibroblasts pre-labelled with DiO were used in experiment 'c', and SAOS-2 were pre-labelled with DiD in that experiment. Results were generally consistent with those described in the main text of the paper.

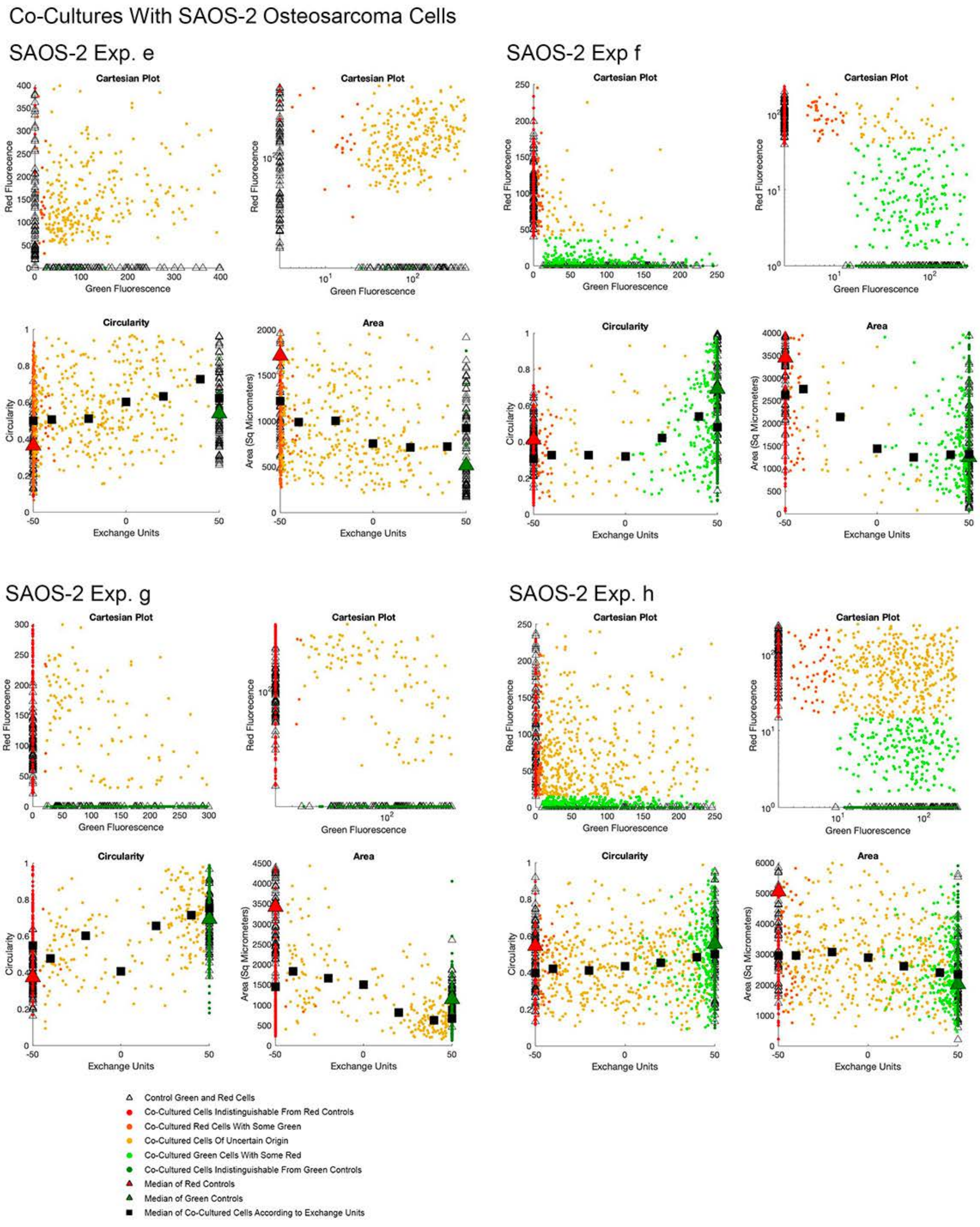

Figure S2. Graphical output of MATLAB script for Cartesian plot analysis of co-cultures of fibroblasts with SAOS-2 osteosarcoma cells in experiments 'e', 'f', 'g' and 'h'. Human dermal fibroblasts were pre-labelled with DiD and SAOS-2 were pre-labelled with DiO. Results were generally consistent with those described in the main text of the paper.

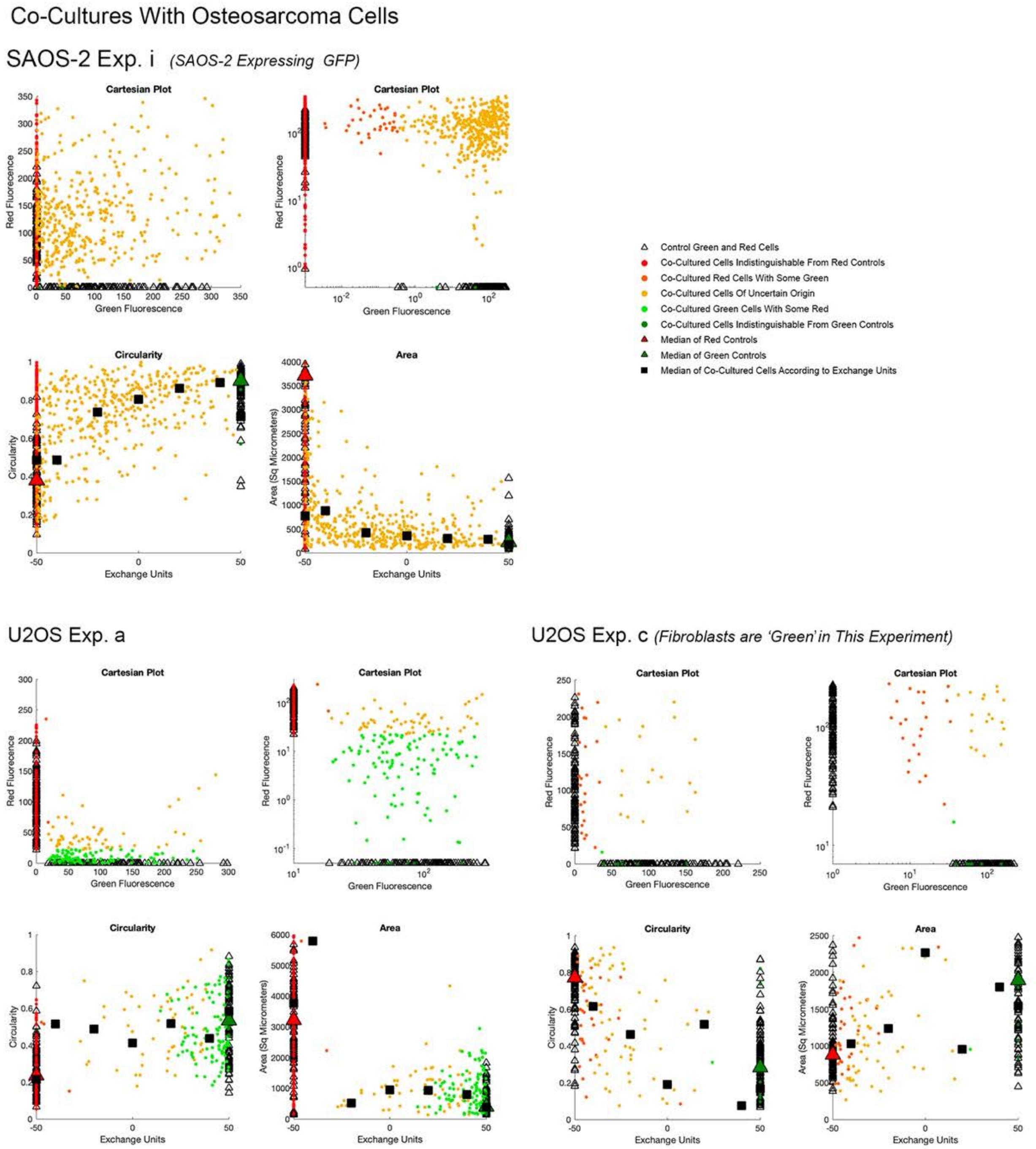

Figure S3. Graphical output of MATLAB script for Cartesian plot analysis of co-cultures of fibroblasts with osteosarcoma cell lines SAOS-2 in experiment 'i', and osteosarcoma U2OS cells in experiments 'a' and 'c'. Human dermal fibroblasts pre-labelled with DiD were used in experiments 'a' and 'i', while human gingival fibroblasts labelled with DiO were used in experiment 'c'. SAOS-2 expressed GFP in experiment 'i', while U2OS were pre-labelled with DiO in experiment 'a', and DiD in experiment 'c'. Results were generally consistent with those described in the main text of the paper.

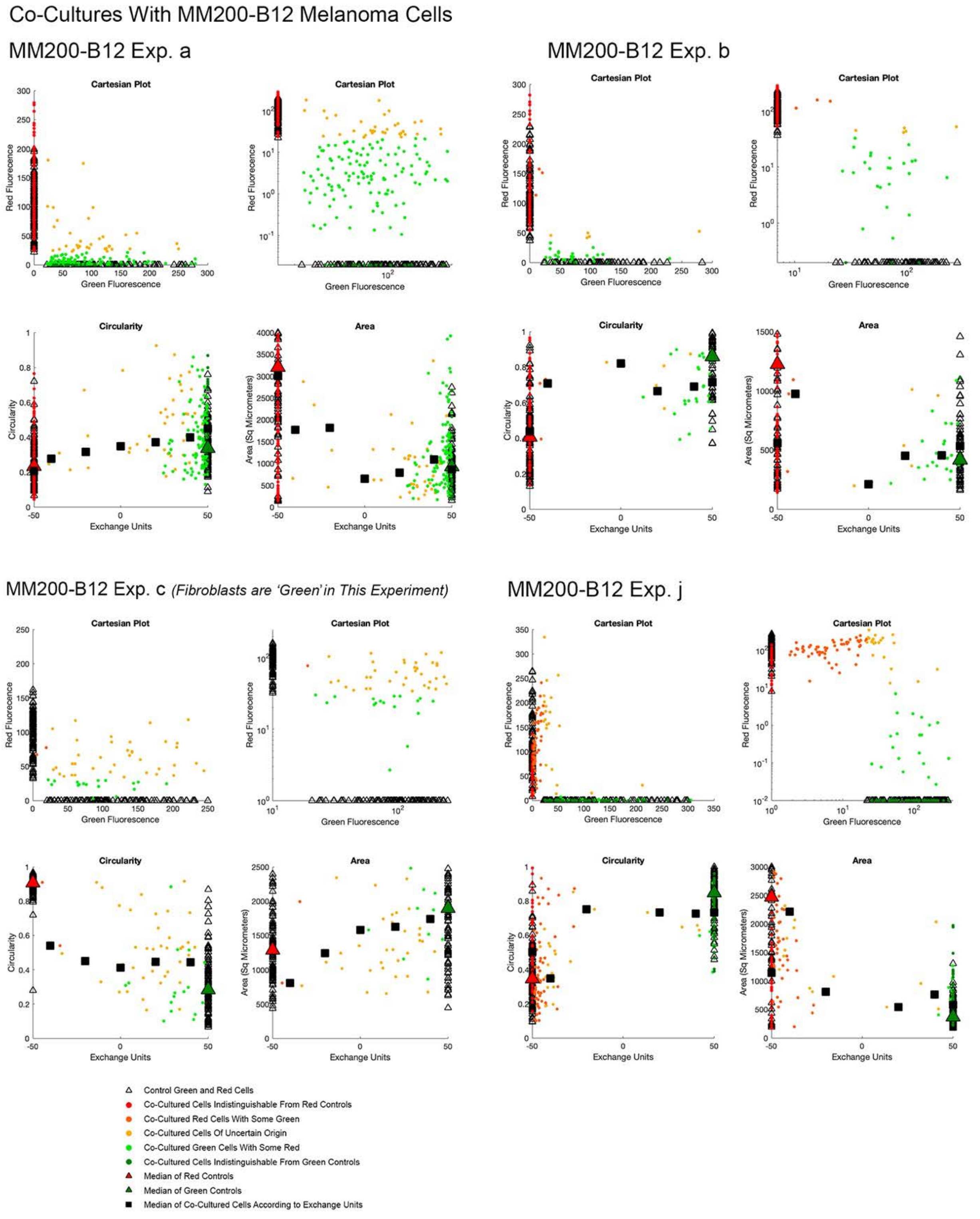

Figure S4. Graphical output of MATLAB script for Cartesian plot analysis of co-cultures of fibroblasts with MM200-B12 melanoma cells in experiments 'a', 'b', 'c' and 'e'. Human dermal fibroblasts pre-labelled with DiD were used in experiments 'a', 'b', and 'j', while MM200-B12 cells were labelled with DiO in these experiments. In experiment 'c', human gingival fibroblasts were used and labelled with DiO, while DiD was used to label MM200-B12 cells in that experiment. Results were generally consistent with those described in the main text of the paper.

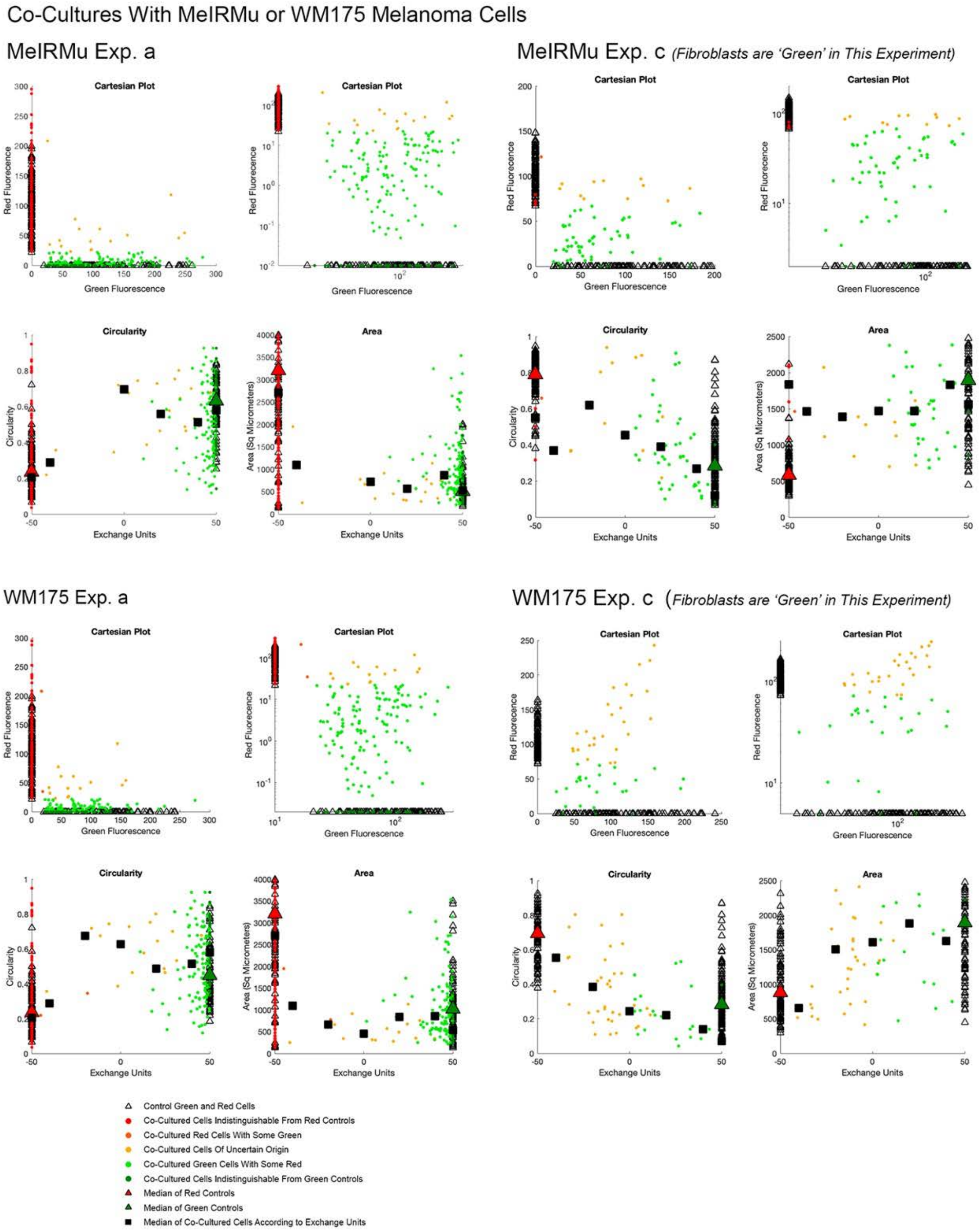

Figure S5. Graphical output of MATLAB script for Cartesian plot analysis of co-cultures of fibroblasts with MeIRMu, and WM175 melanoma cells in experiments 'a' and 'c'. Human dermal fibroblasts pre-labelled with DiD were used in experiment 'a', and melanoma cells were pre-labelled with DiO in that experiment. Human gingival fibroblasts pre-labelled with DiO were used in experiment 'c', while melanoma cells were pre-labelled with DiD in that instance. Results were generally consistent with those described in the main text of the paper.

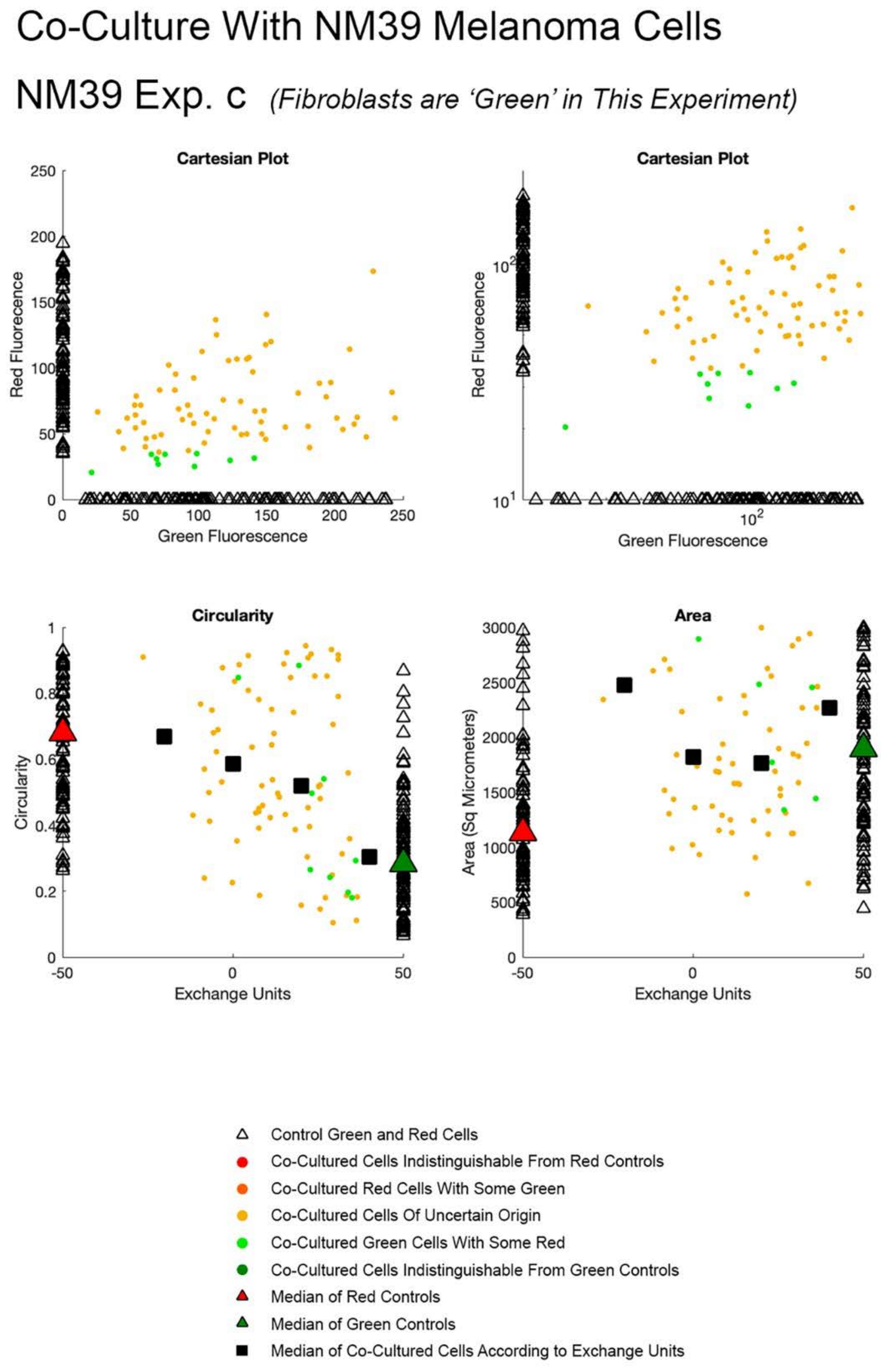

Figure S6. Graphical output of MATLAB script for Cartesian plot analysis of a co-culture of human gingival fibroblasts WM175 melanoma cells in experiment 'c'. Fibroblasts were pre-labelled with DiO, while WM175 cells were pre-labelled with DiD. Results were generally consistent with those described in the main text of the paper.

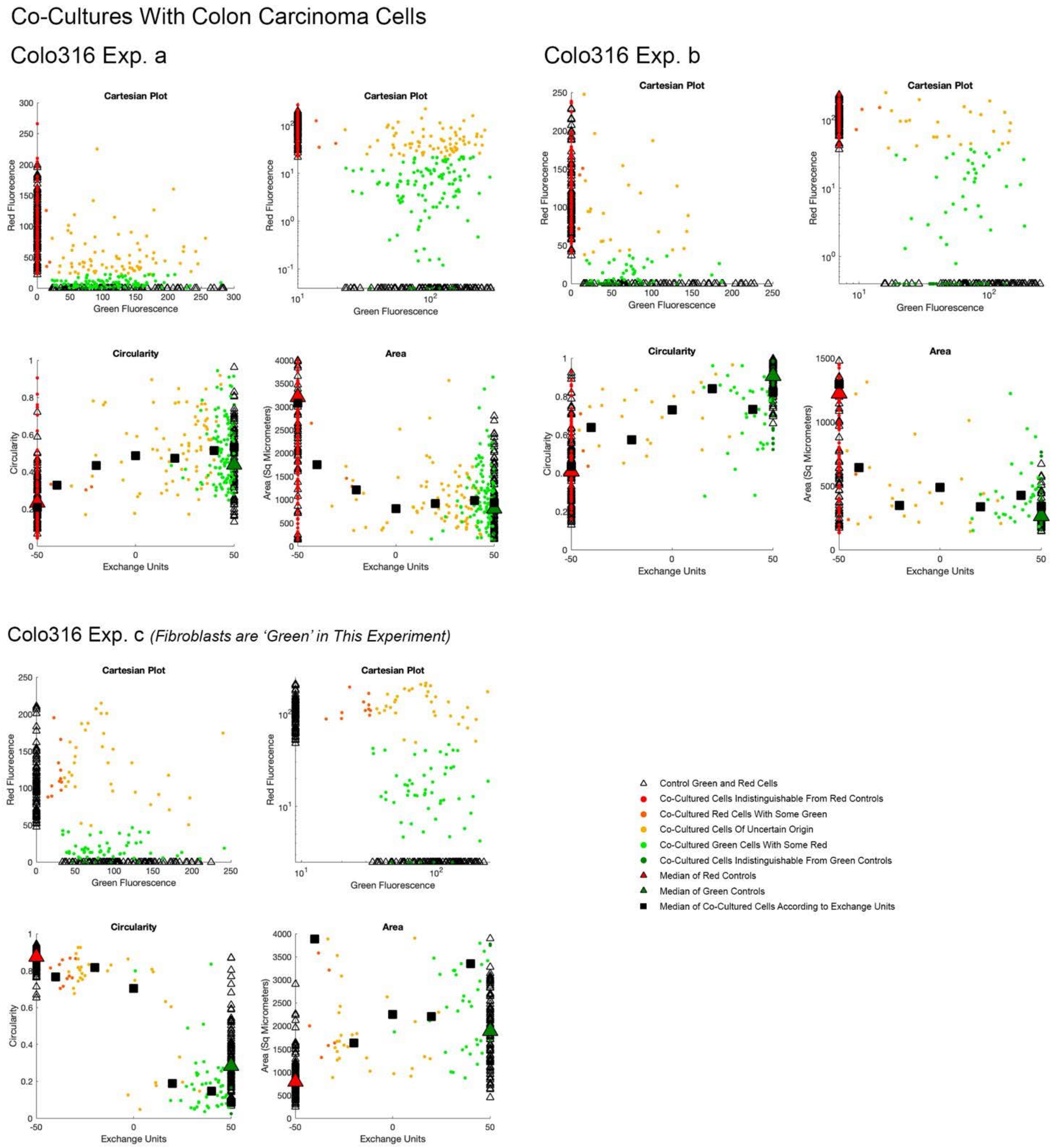

Figure S7. Graphical output of MATLAB script for Cartesian plot analysis of co-cultures of fibroblasts with Colo316 colon carcinoma cells in experiments 'a', 'b', 'c' and 'd'. Human dermal fibroblasts pre-labelled with DiD were used in experiments 'a', 'b' and 'c'. In experiments 'a' and 'b', human dermal fibroblasts pre-labelled with DiD were used, while Colo316 cells were prelabelled with DiO. In experiment 'c', human gingival fibroblasts prelabelled with DiO were used, while Colo316 cells were pre-labelled with DiD. Results were generally consistent with those described in the main text of the paper.

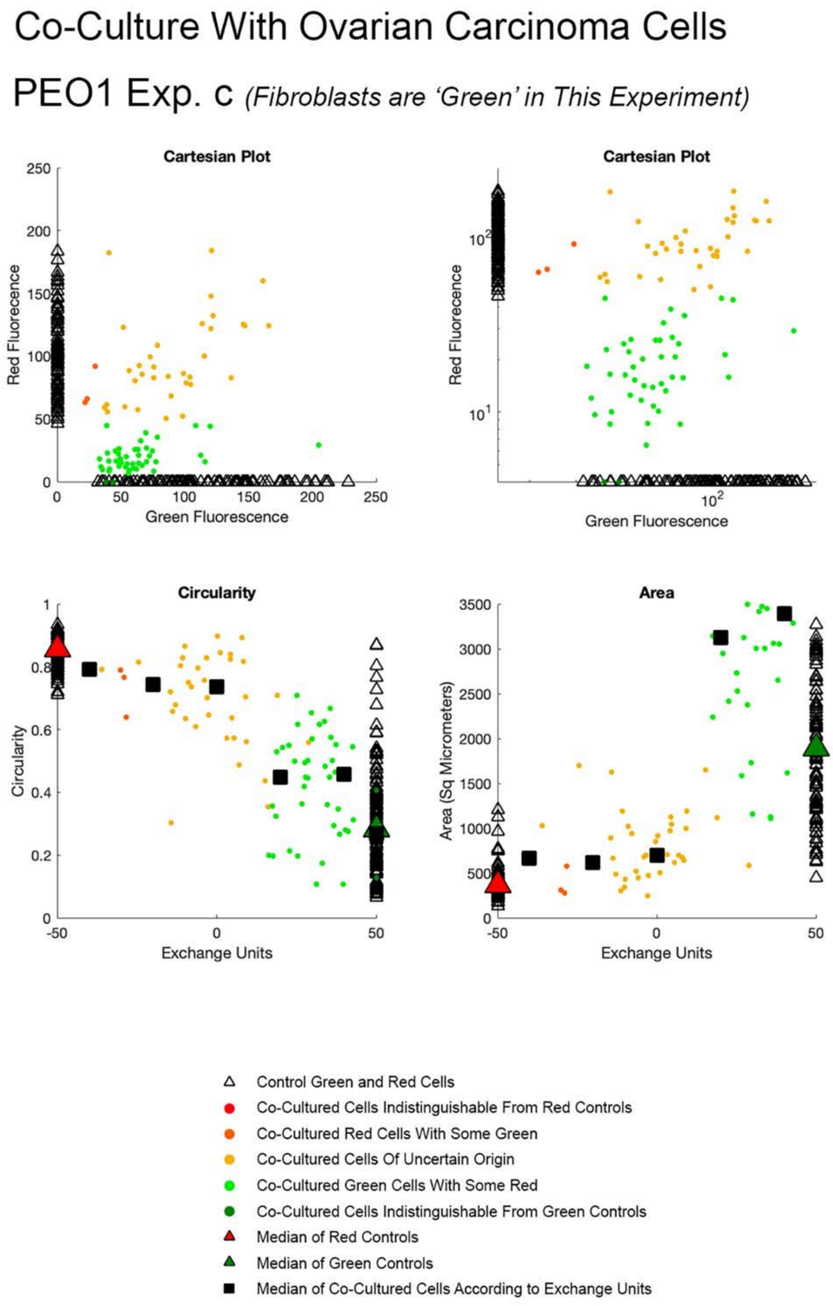

Figure S8. Graphical output of MATLAB script for Cartesian plot analysis of co-culture of DiO pre-labelled human gingival fibroblasts with DiD pre-labelled PEO1 ovarian cancer cells, in experiment 'c'. Results were generally consistent with those described in the main text of the paper.

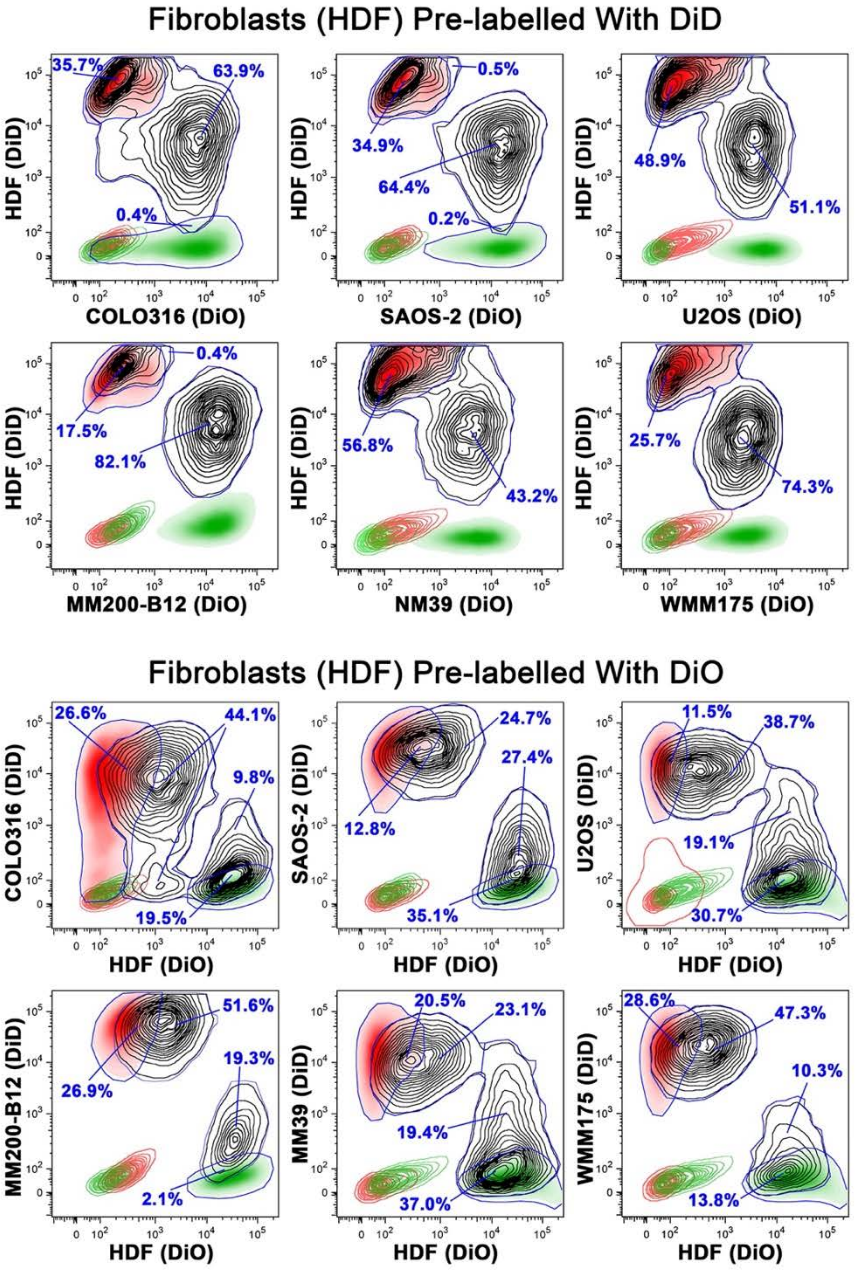

Figure S9. FACS plots of cancer cell lines (Colo316, SAOS-2, U2OS, MM200-B12, NM39, WM175) and human dermal fibroblasts (HDF) pre-labelled with both possible dye orientations of DiD and DiO in experiments 'k' and 'l'. Separate FACS plots for isolated cell culture with and without controls are overlaid with FACS plots for co-cultures. Control cells in isolated culture labelled with DiO are indicated by green shading, while unlabelled control cells of the same type are indicated by green contours. Control cells in isolated culture labelled with DiD are indicated by red shading, and unlabelled controls of the same cell type are indicated by red contours. Co-cultures are marked by black contours. Blue lines indicate gates drawn for co-cultures quantitating the relative percentage of co-cultured cells that have fluorescence indistinguishable from DiD and DiO controls, or have fluorescence shifted beyond those of controls by uptake of fluorescence from the opposing cell type. There was consistent transfer from fibroblasts to cancer cells, irrespective if fibroblasts were pre-labelled with DiD or DiO. Significant transfer from cancer cells to fibroblasts was only seen when cancer cells were pre-labelled with DiD. Data were consistent with Cartesian plot analyses, and also with cell-projection pumping. These results also indicate preferential transfer of DiD over DiO, consistent with high lateral mobility of DiD in membranes made confluent during cell-projection pumping.

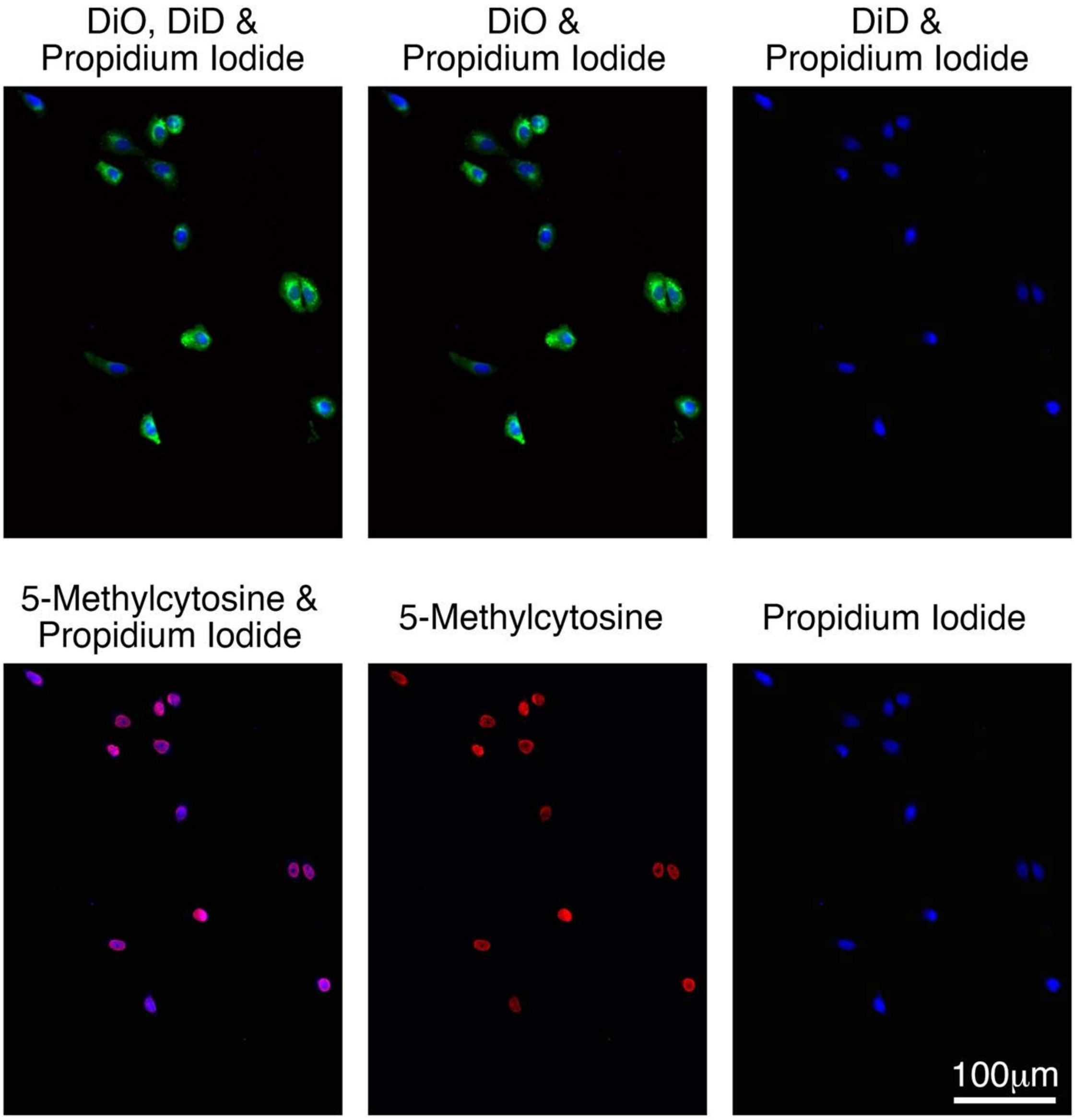

Figure S10. Photomicrographs from experiment 'p' of control SAOS-2 pre-labelled with DiO and cultured in isolation for 24h, and then further labelled with propidium iodide for DNA and by fluorescence immunohistochemistry for 5mc. Channels for DiO, DiD, 5mc and propidium iodide are shown. DiO labelling was as previously seen in experiments 'a' to 'j'. Propidium iodide localized to nuclei, consistent with the intended labelling of DNA. While 5mc fluorescence was in nuclei, 5mc labelling was less uniformly distributed compared with that for propidium iodide, and particularly associated with chromatin at the peripheries of nuclei.

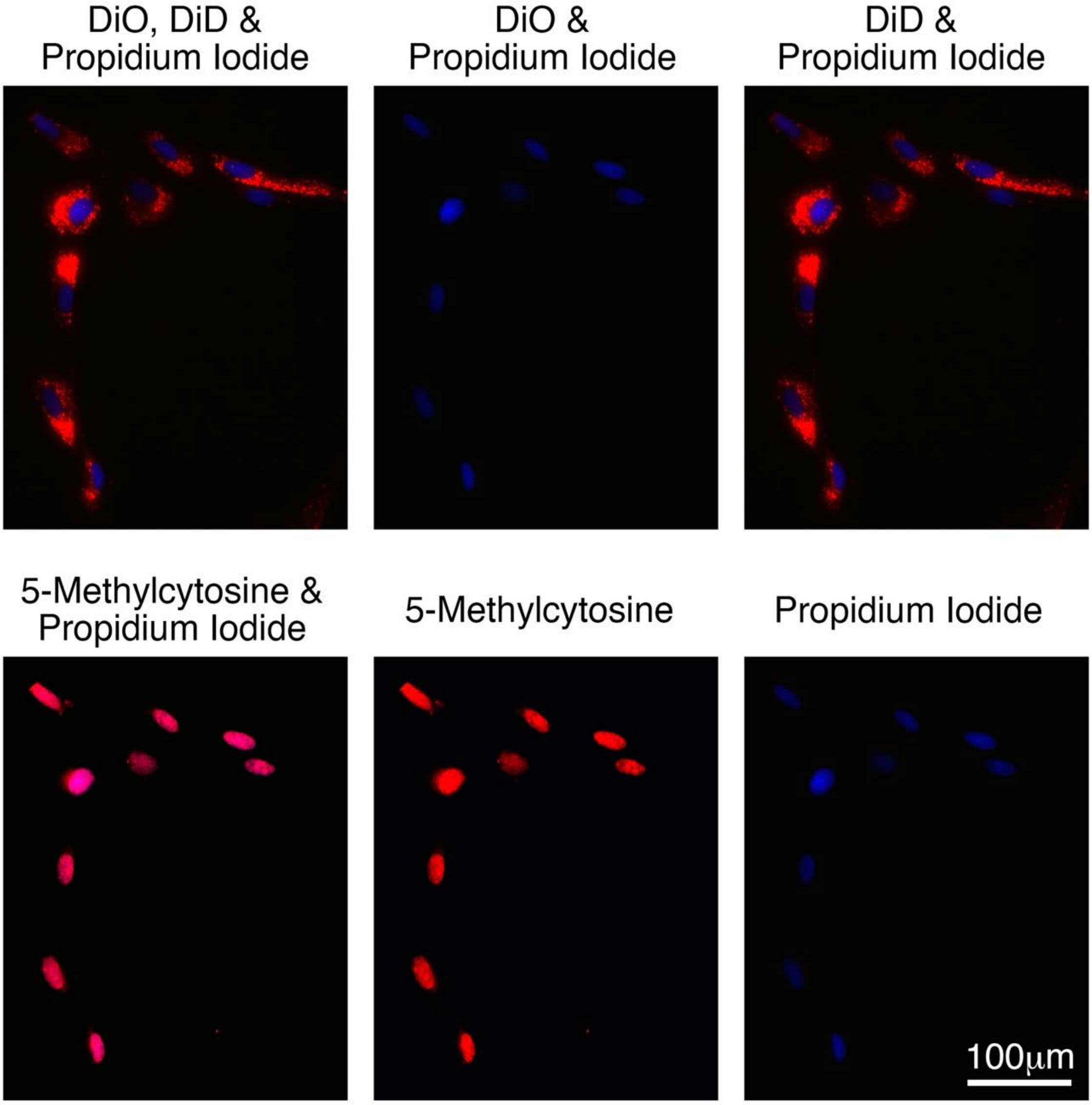

Figure S11. Photomicrographs from experiment 'p' of control human dermal fibroblasts pre-labelled with DiD and cultured in isolation for 24h, and then further labelled with propidium iodide for DNA and by fluorescence immunohistochemistry for 5mc. Channels for DiO, DiD, 5mc and propidium iodide are shown. DiD labelling was as previously seen in experiments 'a' to 'j'. Propidium iodide localized to nuclei, consistent with the intended labelling of DNA. While 5mc fluorescence was in nuclei, 5mc labelling was less uniformly distributed compared with that for propidium iodide. Visual comparison of propidium iodide labelling with that in control SAOS-2 (Figure S10), suggested that fibroblasts had lower levels of DNA compared with SAOS-2. 5mc also appeared more intensely labelled in many fibroblast nuclei than seemed the case in SAOS-2 (Figure S10).

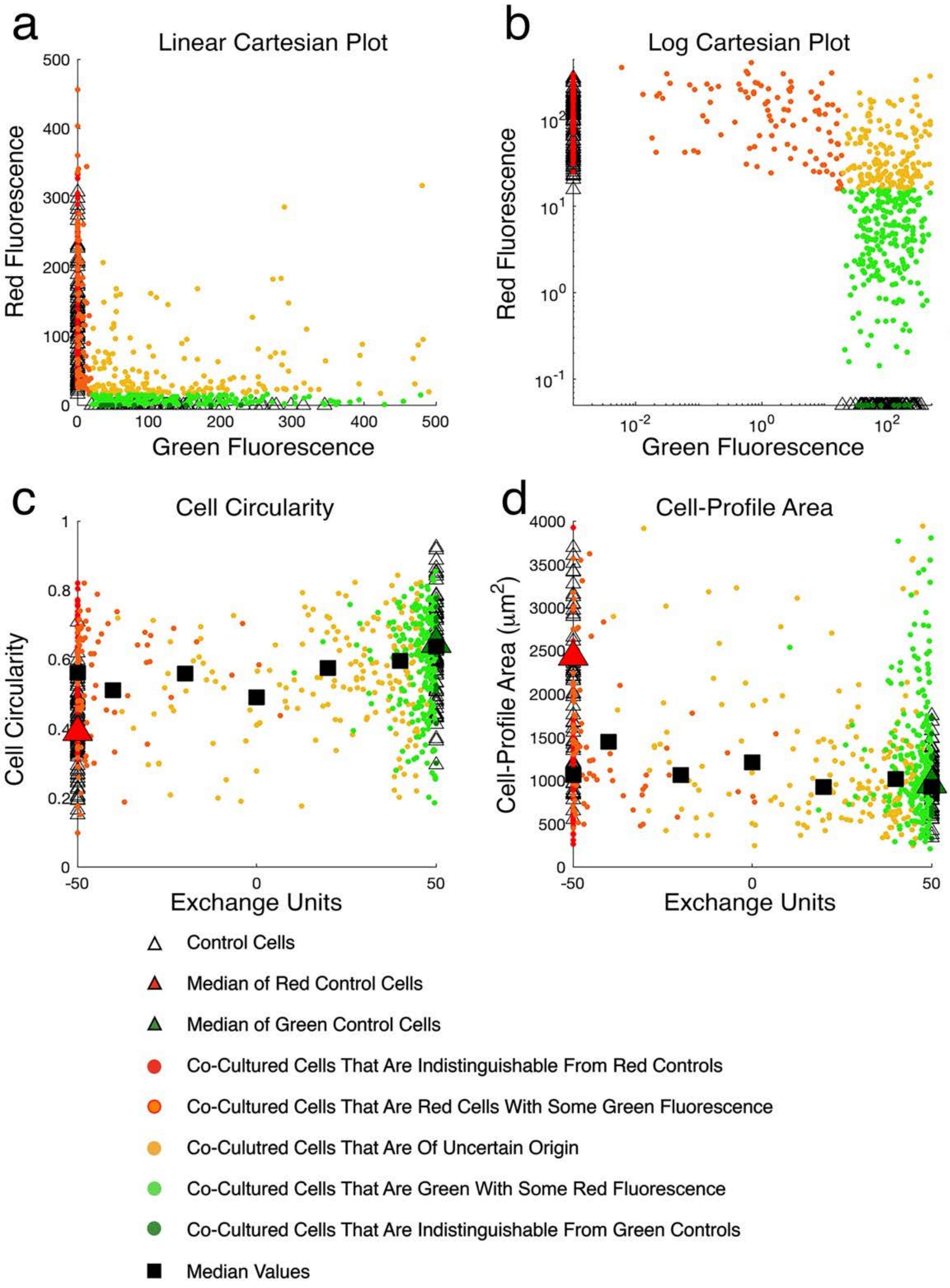

Figure S12. Cartesian plot analysis results for experiment 'n' for cell circularity and cell-profile area where global DNA methylation was separately studied. 'Red Cells' comprise human dermal fibroblasts in these plots, while 'Green Cells' are SAOS-2. (a) Transfer of fluorescence typical of cell-projection pumping resulted in the appearance of cells from all five Cartesian plot groups. Although all five groups are represented in the co-culture shown, they were not present in all of the four co-cultures where DNA methylation was studied ('m', 'o', 'p'). Control human dermal fibroblasts cultured in isolation had lower cell circularity compared with SAOS-2 controls ($p < 0.0001$, Mann Whitney U test), as well as higher cell-profile area ($p < 0.0001$, Mann Whitney U Test). This difference was maintained in co-cultures considering cells with fluorescence indistinguishable from controls, but did not reach statistical significance in this co-culture, as detailed in Tables S25 to S28 . The general pattern of data was similar to that described for co-cultures 'a' to 'j'.

Supplementary Tables

Page	Table
17	**Table S1.** Median values for cell circularity for control and co-cultured cells in experiments 'a' to 'j' according to Cartesian Plot classification, and statistical significance of differences between these as per the Wilcoxon Signed Rank Test.
17	**Table S2.** Median values for cell circularity for control and co-cultured cells in experiments 'a' to 'i' with SAOS-2 according to Cartesian Plot classification, and statistical significance of differences between these as per the Wilcoxon Signed Rank Test.
18	**Table S3.** Median values for cell-profile area for control and co-cultured cells in experiments 'a' to 'j' according to Cartesian Plot classification, and statistical significance of differences between these as per the Wilcoxon Signed Rank Test.
18	**Table S4.** Median values for cell-profile area for control and co-cultured cells in experiments 'a' to 'i' with SAOS-2 according to Cartesian Plot classification, and statistical significance of differences between these as per the Wilcoxon Signed Rank Test.
19	**Table S5.** Median values for cell circularity for control and co-cultured cells in experiments 'a' to 'j' according to Exchange Units (EU), and statistical significance of differences between these as per the Wilcoxon Signed Rank Test.
19	**Table S6.** Median values for cell circularity for control and co-cultured cells in experiments 'a' to 'i' with SAOS-2 according to Exchange Units (EU), and statistical significance of differences between these as per the Wilcoxon Signed Rank Test.
20	**Table S7.** Median values for cell-profile area for control and co-cultured cells in experiments 'a' to 'j' according to Exchange Units (EU), and statistical significance of differences between these as per the Wilcoxon Signed Rank Test.
20	**Table S8.** Median values for cell-profile area for control and co-cultured cells in experiments 'a' to 'i' with SAOS-2 according to Exchange Units (EU), and statistical significance of differences between these as per the Wilcoxon Signed Rank Test.
21	**Table S9.** Statistical significance regarding fundamental differences in cell circularity and cell-profile area between cancer cells and fibroblasts cultured in isolation or together, for experiments 'a' to 'j'.
22	**Table S10.** Numerical summary of results shown in Table S9 of the statistics relating to fundamental differences in cell circularity and cell-profile area between cancer cells and fibroblasts cultured in isolation or together, for experiments 'a' to 'j'.
23	**Table S11.** Statistical significance regarding phenotypic association between cell circularity and classification of cells according to Cartesian plot group, in experiments 'a' to 'j'.

24 **Table S12.** Statistical significance regarding phenotypic association between cell circularity and Exchange Units (EU), in experiments 'a' to 'j'.

25 **Table S13.** Numerical summary of results shown in Tables S11 and S12 of the statistics relating to cell circularity in experiments 'a' to 'j'.

26 **Table S14.** Statistical significance regarding phenotypic association between cell-profile area and classification of cells according to Cartesian plot group, in experiments 'a' to 'j'.

27 **Table S15.** Statistical significance regarding phenotypic association between cell-profile area and Exchange Units (EU), in experiments 'a' to 'j'.

28 **Table S16.** Numerical summary of results shown in Tables S14 and S15 of the statistics relating to cell-profile area in experiments 'a' to 'j'.

29 **Table S17.** Median values for cell-profile area in control cells cultured in isolation, as well as in co-cultured cells according to Cartesian Plot grouping in each experiment for assessment of global DNA methylation (experiments 'm' to 'p').

30 **Table S18.** Median values for cell circularity in control cells cultured in isolation, as well as in co-cultured cells according to Cartesian Plot grouping in each experiment for assessment of global DNA methylation (experiments 'm' to 'p').

31 **Table S19.** Median values for cell-profile area in control cells cultured in isolation, as well as in co-cultured cells according to EU grouping in each experiment for assessment of global DNA methylation (experiments 'm' to 'p').

32 **Table S20.** Median values for cell circularity in control cells cultured in isolation, as well as in co-cultured cells according to EU grouping in each experiment for assessment of global DNA methylation (experiments 'm' to 'p').

33 **Table S21.** Median values for normalized 5mc in control cells cultured in isolation, as well as in co-cultured cells according to Cartesian Plot grouping, in each experiment for assessment of global DNA methylation (experiments 'm' to 'p').

34 **Table S22.** Median values for normalized 5mc in control cells cultured in isolation, as well as in co-cultured cells according to EU grouping, in each experiment for assessment of global DNA methylation (experiments 'm' to 'p').

Table S1. Median values for cell circularity for control and co-cultured cells in experiments 'a' to 'j' according to Cartesian Plot classification, and statistical significance of differences between these as per the Wilcoxon Signed Rank Test. The medians shown are of the separate medians from individual experiments. Statistical significance was accepted at $p < 0.05$, and the absence of statistical significance between median values is indicated by 'NS' for 'not significant'.

			Co-Cultured Cells					
		Median Values for Cell Circularity	Cells Indistinguishable From Cancer Cell Controls	Cancer Cells With Some Fibroblast Labelling	Cells of Uncertain Origin	Fibroblasts with Some Cancer Cell Labelling	Cells Indistinguishable From Fibroblast Controls	Control Fibroblasts
	Median Values for Cell Circularity		0.582	0.5295	0.559	0.342	0.2675	0.283
			Statistical Significance of Differences Between Medians					
	Control Cancer Cells	0.733	NS	0.0001	0.0018	<0.0001	<0.0001	<0.0001
Co-Cultured Cells	Cells Indistinguishable From Cancer Cell Controls	0.582		NS	NS	<0.0001	<0.0001	<0.0001
Co-Cultured Cells	Cancer Cells With Some Fibroblast Labelling	0.529			NS	0.0006	<0.0001	<0.0001
Co-Cultured Cells	Cells of Uncertain Origin	0.559				<0.0001	<0.0001	<0.0001
Co-Cultured Cells	Fibroblasts with Some Cancer Cell Labelling	0.342					0.0296	NS
Co-Cultured Cells	Cells Indistinguishable From Fibroblast Controls	0.267						NS

Table S2. Median values for cell circularity for control and co-cultured cells in experiments 'a' to 'i' with SAOS-2 according to Cartesian Plot classification, and statistical significance of differences between these as per the Wilcoxon Signed Rank Test. The medians shown are of the separate medians from individual experiments. Statistical significance was accepted at $p < 0.05$, and the absence of statistical significance between median values is indicated by 'NS' for 'not significant'.

			Co-Cultured Cells					
		Median Values for Cell Circularity	Cells Indistinguishable From SAOS-2 Controls	SAOS-2 With Some Fibroblast Labelling	Cells of Uncertain Origin	Fibroblasts with Some SAOS-2 Labelling	Cells Indistinguishable From Fibroblast Controls	Control Fibroblasts
	Median Values for Cell Circularity		0.6236	0.4876	0.573	0.3962	0.4516	0.3727
			Statistical Significance of Differences Between Medians					
	Control SAOS-2	0.6919	NS	NS	0.0117	0.0078	0.0078	0.0039
Co-Cultured Cells	Cells Indistinguishable From SAOS-2 Controls	0.6236		NS	NS	0.0078	0.0078	0.0078
Co-Cultured Cells	SAOS-2 With Some Fibroblast Labelling	0.4876			NS	NS	NS	NS
Co-Cultured Cells	Cells of Uncertain Origin	0.573				0.0391	0.0078	0.0391
Co-Cultured Cells	Fibroblasts with Some SAOS-2 Labelling	0.3962					NS	NS
Co-Cultured Cells	Cells Indistinguishable From Fibroblast Controls	0.4516						NS

Table S3. Median values for cell-profile area for control and co-cultured cells in experiments 'a' to 'j' according to Cartesian Plot classification, and statistical significance of differences between these as per the Wilcoxon Signed Rank Test. The medians shown are of the separate medians from individual experiments. Statistical significance was accepted at $p < 0.05$, and the absence of statistical significance between median values is indicated by 'NS' for 'not significant'.

		Median Values for Cell-Profile Area (μm^2)	Co-Cultured Cells: Cells Indistinguishable From Cancer Cell Controls	Co-Cultured Cells: Cancer Cells With Some Fibroblast Labelling	Co-Cultured Cells: Cells of Uncertain Origin	Co-Cultured Cells: Fibroblasts with Some Cancer Cell Labelling	Co-Cultured Cells: Cells Indistinguishable From Fibroblast Controls	Control Fibroblasts
	Median Values for Cell- Profile Area (μm^2)		753	1,060	932	1,641	2,619	2,181
			Statistical Significance of Differences Between Medians					
	Control Cancer Cells	713	NS	0.0001	0.0002	<0.0001	<0.0001	<0.0001
Co-Cultured Cells	Cells Indistinguishable From Cancer Cell Controls	753		0.0419	NS	0.0002	<0.0001	<0.0001
Co-Cultured Cells	Cancer Cells With Some Fibroblast Labelling	1,060			NS	0.0015	<0.0001	0.0003
Co-Cultured Cells	Cells of Uncertain Origin	932				<0.0001	<0.0001	<0.0001
Co-Cultured Cells	Fibroblasts with Some Cancer Cell Labelling	1,641					NS	NS
Co-Cultured Cells	Cells Indistinguishable From Fibroblast Controls	2,619						NS

Table S4. Median values for cell-profile area for control and co-cultured cells in experiments 'a' to 'i' with SAOS-2 according to Cartesian Plot classification, and statistical significance of differences between these as per the Wilcoxon Signed Rank Test. The medians shown are of the separate medians from individual experiments. Statistical significance was accepted at $p < 0.05$, and the absence of statistical significance between median values is indicated by 'NS' for 'not significant'.

		Median Values for Cell-Profile Area (μm^2)	Co-Cultured Cells: Cells Indistinguishable From SAOS-2 Controls	Co-Cultured Cells: SAOS-2 With Some Fibroblast Labelling	Co-Cultured Cells: Cells of Uncertain Origin	Co-Cultured Cells: Fibroblasts with Some SAOS-2 Labelling	Co-Cultured Cells: Cells Indistinguishable From Fibroblast Controls	Control Fibroblasts
	Median Values for Cell- Profile Area (μm^2)		875	1,325	993	1,954	1,357	3,417
			Statistical Significance of Differences Between Medians					
	Control SAOS-2	636	NS	NS	0.0117	0.0156	0.0078	0.0039
Co-Cultured Cells	Cells Indistinguishable From SAOS-2 Controls	875		NS	NS	0.0078	0.0078	0.0039
Co-Cultured Cells	SAOS-2 With Some Fibroblast Labelling	1,325			NS	NS	NS	NS
Co-Cultured Cells	Cells of Uncertain Origin	993				0.0156	0.0078	0.0039
Co-Cultured Cells	Fibroblasts with Some SAOS-2 Labelling	1,954					NS	NS
Co-Cultured Cells	Cells Indistinguishable From Fibroblast Controls	1,357						0.0391

Table S5. Median values for cell circularity for control and co-cultured cells in experiments 'a' to 'j' according to Exchange Units (EU), and statistical significance of differences between these as per the Wilcoxon Signed Rank Test. The medians shown are of the separate medians from individual experiments. Statistical significance was accepted at $p < 0.05$, and the absence of statistical significance between median values is indicated by 'NS' for 'not significant'.

			Co-Cultured Cells							
		Median Values for Cell Circularity	EU = 50	30 < EU <50	10 < EU ≤ 30	-10 < EU ≤ 10	-30 < EU ≤ -10	-50 < EU ≤ -30	EU = -50	Control Fibroblasts
	Median Values for Cell Circularity		0.582	0.553	0.5887	0.4697	0.4868	0.347	0.2675	0.283
			Statistical Significance of Differences Between Medians							
	Control Cancer Cells	0.7333	NS	0.0024	0.0004	0.0007	0.0003	<0.0001	<0.0001	<0.0001
Co-Cultured Cells	EU = 50	0.582		NS	NS	NS	0.0151	<0.0001	<0.0001	<0.0001
	30 < EU <50	0.553			NS	0.0421	0.0064	<0.0001	<0.0001	<0.0001
	10 < EU ≤ 30	0.5887				0.0172	0.008	<0.0001	<0.0001	<0.0001
	-10 < EU ≤ 10	0.4697					NS	0.0003	<0.0001	<0.0001
	-30 < EU ≤ -10	0.4868						0.0014	<0.0001	0.0002
	-50 < EU ≤ -30	0.347							0.0081	0.0475
	EU = -50	0.2675								NS

Table S6. Median values for cell circularity for control and co-cultured cells in experiments 'a' to 'i' with SAOS-2 according to Exchange Units (EU), and statistical significance of differences between these as per the Wilcoxon Signed Rank Test. The medians shown are of the separate medians from individual experiments. Statistical significance was accepted at $p < 0.05$, and the absence of statistical significance between median values is indicated by 'NS' for 'not significant'.

			Co-Cultured Cells							
		Median Values for Cell Circularity	EU = 50	30 < EU <50	10 < EU ≤ 30	-10 < EU ≤ 10	-30 < EU ≤ -10	-50 < EU ≤ -30	EU = -50	Control Fibroblasts
	Median Values for Cell Circularity		0.6236	0.713	0.6319	0.4377	0.5564	0.4491	0.4516	0.3727
			Statistical Significance of Differences Between Medians							
	Control SAOS-2	0.6919	NS	NS	0.0391	0.0156	0.0391	0.0078	0.0078	0.0039
Co-Cultured Cells	EU = 50	0.6236		NS	NS	NS	NS	0.0078	0.0078	0.0078
	30 < EU <50	0.713			NS	0.0078	NS	0.0078	0.0078	0.0078
	10 < EU ≤ 30	0.6319				0.0078	NS	0.0078	0.0078	0.0195
	-10 < EU ≤ 10	0.4377					NS	NS	NS	NS
	-30 < EU ≤ -10	0.5564						NS	0.0156	0.0781
	-50 < EU ≤ -30	0.4491							NS	NS
	EU = -50	0.4516								NS

Table S7. Median values for cell-profile area for control and co-cultured cells in experiments 'a' to 'j' according to Exchange Units (EU), and statistical significance of differences between these as per the Wilcoxon Signed Rank Test. The medians shown are of the separate medians from individual experiments. Statistical significance was accepted at $p < 0.05$, and the absence of statistical significance between median values is indicated by 'NS' for 'not significant'.

			Co-Cultured Cells							
		Median Values for Cell-Profile Area (μm^2)	EU = 50	30 < EU <50	10 < EU ≤ 30	-10 < EU ≤ 10	-30 < EU ≤ -10	-50 < EU ≤ -30	EU = -50	Control Fibroblasts
	Median Values for Cell-Profile Area (μm^2)		753	853	878	980	1,466	1,766	2,619	2,181
			Statistical Significance of Differences Between Medians							
	Control Cancer Cells	713	NS	0.0091	0.0008	0.0015	<0.0001	<0.0001	<0.0001	<0.0001
Co-Cultured Cells	EU = 50	753		0.0304	NS	NS	0.002	<0.0001	<0.0001	<0.0001
	30 < EU <50	853			NS	NS	NS	<0.0001	<0.0001	<0.0001
	10 < EU ≤ 30	878				NS	0.0239	<0.0001	<0.0001	<0.0001
	-10 < EU ≤ 10	980					0.0328	<0.0001	0.0001	<0.0001
	-30 < EU ≤ -10	1,466						0.0003	<0.0001	0.0001
	-50 < EU ≤ -30	1,766							NS	NS
	EU = -50	2,619								NS

Table S8. Median values for cell-profile area for control and co-cultured cells in experiments 'a' to 'i' with SAOS-2 according to Exchange Units (EU), and statistical significance of differences between these as per the Wilcoxon Signed Rank Test. The medians shown are of the separate medians from individual experiments. Statistical significance was accepted at $p < 0.05$, and the absence of statistical significance between median values is indicated by 'NS' for 'not significant'.

			Co-Cultured Cells							
		Median Values for Cell-Profile Area (μm^2)	EU = 50	30 < EU <50	10 < EU ≤ 30	-10 < EU ≤ 10	-30 < EU ≤ -10	-50 < EU ≤ -30	EU = -50	Control Fibroblasts
	Median Values for Cell-Profile Area (μm^2)		875	925	811	1,062	1,413	1,702	1,357	3,417
			Statistical Significance of Differences Between Medians							
	Control SAOS-2	636	NS	NS	NS	0.0078	0.0078	0.0078	0.0078	0.0039
Co-Cultured Cells	EU = 50	875		NS	NS	NS	0.0078	0.0078	0.0078	0.0039
	30 < EU <50	925			NS	NS	NS	0.0078	0.0078	0.0039
	10 < EU ≤ 30	811				NS	NS	0.0078	0.0078	0.0039
	-10 < EU ≤ 10	1,062					0.0078	0.0078	0.0312	0.0078
	-30 < EU ≤ -10	1,413						NS	NS	0.0078
	-50 < EU ≤ -30	1,702							NS	NS
	EU = -50	1,357								0.0391

Table S9. Statistical significance regarding fundamental differences in cell circularity and cell-profile area between cancer cells and fibroblasts cultured in isolation or together, for experiments 'a' to 'j'. Observations are described as statements numbered 1 to 8, marked 'T' for 'True' or 'F' for 'False', by comparison of median values within experiments. Statistical significance is indicated in instances where this was reached at $p < 0.05$ by Mann Whitney U Test, for both 'T' and 'F' results. NS indicates that the results was 'not statistically significant. In some co-cultures, cancer cells or fibroblasts with fluorescence identical to that of control cells were absent, so that comparison was not possible, and this is reflected by the presence of 'blank' cells. Table S10 gives a numerical summary of results in this table.

Statements On Experimental Results

1. Circularity of control fibroblasts cultured alone was less than in control cancer cells cultured alone
2. Circularity of control fibroblasts cultured alone was less than in co-cultured cancer cells with fluorescence indistinguishable from controls
3. Circularity of co-cultured fibroblasts with fluorescence indistinguishable from controls was less than in control cancer cells cultured alone
4. Circularity of co-cultured fibroblasts with fluorescence indistinguishable from controls was less than in co-cultured cancer cells with fluorescence indistinguishable from controls
5. Cell-profile area of control fibroblasts cultured alone was greater than in control cancer cells cultured alone
6. Cell-profile area of control fibroblasts cultured alone was greater than in co-cultured cancer cells with fluorescence indistinguishable from controls
7. Cell-profile area of co-cultured fibroblasts with fluorescence indistinguishable from controls was greater than in control cancer cells cultured alone
8. Cell-profile area of co-cultured fibroblasts with fluorescence indistinguishable from controls was greater than in co-cultured cancer cells with fluorescence indistinguishable from controls

		Statistical Significance According to Observation Number							
Cell Line	Exp.	1	2	3	4	5	6	7	8
SAOS-2	a	T, <0.0001	T, NS	T, <0.0001	T, NS	T, <0.0001	T, NS	T, <0.0001	T, NS
SAOS-2	b	T, <0.0001	T, <0.0001	T, <0.0001	T, <0.0001	T, 0.0023	T, 0.0002	T, 0.0138	T, 0.0027
SAOS-2	c	T, <0.0001	T, NS			T, <0.0001	T, 0.0068		
SAOS-2	d	T, <0.0001	T, <0.0001	T, <0.0001	T, <0.0001	T, <0.0001	T, <0.0001	T, <0.0001	T, <0.0001
SAOS-2	e	T, <0.0001	T, <0.0001	T, 0.0104	T, 0.0240	T, <0.0001	T, 0.0194	T, <0.0001	T, 0.0414
SAOS-2	f	T, <0.0001	T, <0.0001	T, <0.0001	T, <0.0001	T, <0.0001	T, <0.0001	T, <0.0001	T, <0.0001
SAOS-2	g	T, <0.0001	T, <0.0001	T, <0.0001	T, <0.0001	T, <0.0001	T, <0.0001	F, NS	T, <0.0001
SAOS-2	h	T, NS	F, NS	T, <0.0001	T, 0.0002	T, <0.0001	T, <0.0001	T, <0.0001	T, 0.0004
SAOS-2	i	T, <0.0001	T, 0.0489	T, <0.0001	T, NS	T, <0.0001	T, 0.0291	T, <0.0001	T, NS
U2OS	a	T, <0.0001	T, <0.0001	T, <0.0001	T, <0.0001	T, <0.0001	T, 0.0061	T, <0.0001	T, <0.0001
U2OS	c	T, <0.0001		T, NS		T, <0.0001		T, NS	
MeIRMu	a	T, <0.0001	T, <0.0001	T, <0.0001	T, <0.0001	T, <0.0001	T, <0.0001	T, <0.0001	T, <0.0001
MeiRMu	c	T, <0.0001	T, NS	T, 0.0092	T, NS	T, <0.0001	T, NS	T, 0.0076	T, NS
MM200-B12	a	T, 0.0007	T, <0.0001	T, 0.0011	T, 0.0001	T, <0.0001	T, 0.0012	T, <0.0001	T, 0.0004
MM200-B12	b	T, <0.0001	T, 0.0082	T, <0.0001	T, NS	T, <0.0001	T, NS	T, 0.0140	F, NS
MM200-B12	c	T, <0.0001				T, 0.0007			
MM200-B12	j	T, <0.0001	T, <0.0001	T, <0.0001	T, <0.0001	T, <0.0001	T, <0.0001	T, <0.0001	T, 0.0008
WM175	a	T, <0.0001	T, <0.0001	T, <0.0001	T, <0.0001	T, <0.0001	T, <0.0001	T, <0.0001	T, <0.0001
WM175	c	T, <0.0001		T, NS		T, <0.0001		T, <0.0001	
NM39	c	T, <0.0001				T, 0.0085			
Colo316	a	T, <0.0001	T, 0.0006	T, <0.0001	T, 0.0001	T, <0.0001	T, NS	T, <0.0001	T, 0.0094
Colo316	b	T, <0.0001	T, <0.0001	T, <0.0001	T, <0.0001	T, <0.0001	T, <0.0001	T, <0.0001	T, 0.0005
Colo316	c	T, <0.0001		T, 0.0055		T, <0.0001		T, <0.0001	
PEO1	c	T, <0.0001		T, 0.0005		T, <0.0001		T, 0.0005	

Interpretation is outlined in Table S10

Table S10. Numerical summary of results shown in Table S9 of the statistics relating to fundamental differences in cell circularity and cell-profile area between cancer cells and fibroblasts cultured in isolation or together, for experiments 'a' to 'j'. Observations were described as statements numbered 1 to 8, and identified as 'True' or 'False' within individual experiments. For both True and False statements, distinction was made if results were statistically significant (Sig.) by Mann Whitney U Test or not (Not Sig.) to $p < 0.05$. In some co-cultures, cancer cells or fibroblasts with fluorescence identical to that of control cells were absent, so that comparison was not possible, and this reduced the number of observations possible (Obs. Poss.). To aid interpretation, the percentage of 'True statements (% Total 'True')', 'True statements that were statistically significant' (% 'True' Sig.) , and 'True statements that were not statistically significant (% 'True' Not Sig.) was calculated and is shown.

Statements On Experimental Results

1. Circularity of control fibroblasts cultured alone was less than in control cancer cells cultured alone
2. Circularity of control fibroblasts cultured alone was less than in co-cultured cancer cells with fluorescence indistinguishable from controls
3. Circularity of co-cultured fibroblasts with fluorescence indistinguishable from controls was less than in control cancer cells cultured alone
4. Circularity of co-cultured fibroblasts with fluorescence indistinguishable from controls was less than in co-cultured cancer cells with fluorescence indistinguishable from controls
5. Cell-profile area of control fibroblasts cultured alone was greater than in control cancer cells cultured alone
6. Cell-profile area of control fibroblasts cultured alone was greater than in co-cultured cancer cells with fluorescence indistinguishable from controls
7. Cell-profile area of co-cultured fibroblasts with fluorescence indistinguishable from controls was greater than in control cancer cells cultured alone
8. Cell-profile area of co-cultured fibroblasts with fluorescence indistinguishable from controls was greater than in co-cultured cancer cells with fluorescence indistinguishable from controls

Statement No.	True, Sig.	True, Not Sig.	False, Sig.	False, Not Sig.	Missing Observations	Total True	Obs. Poss.	% Total 'True'	% 'True' Sig.	% 'True' Not Sig.
1	23	1	0	0	0	24	24	100.0	95.8	4.2
2	14	3	0	1	6	17	18	94.4	77.8	16.7
3	19	2	0	0	3	21	21	100.0	90.5	9.5
4	13	4	0	0	7	17	17	100.0	76.5	23.5
5	24	0	0	0	0	24	24	100.0	100.0	0.0
6	14	4	0	0	6	18	18	100.0	77.8	22.2
7	19	1	0	1	3	20	21	95.2	90.5	4.8
8	13	3	0	1	7	16	17	94.1	76.5	17.6

In almost all co-culture experiments, fibroblasts had lower cell circularity and greater cell-profile area, relative to cancer cells. This was most reproducible when control populations of fibroblasts and co-cultured cells were compared against each other (Statements 1, 2). These distinctions were also seen making further comparisons that included cells in co-culture, but were less often statistically significant and very occasionally absent in individual co-cultures.

Table S11. Statistical significance regarding phenotypic association between cell circularity and classification of cells according to Cartesian plot group, in experiments 'a' to 'j'. Observations are described as statements numbered 1 to 10, marked 'T' for 'True' or 'F' for 'False', by comparison of median values within experiments. Statistical significance is indicated in instances where this was reached at $p < 0.05$ by Mann Whitney U Test. NS indicates that the results was not statistically significant. In a number of co-cultures, some groups were absent and this accounts for 'blank' cells.

Statements On Experimental Results

1. Circularity of co-cultured cancer cells with fluorescence indistinguishable from controls was less than in control cancer cells cultured alone
2. Circularity of co-cultured cancer cells with some fibroblast label was less than in control cancer cells cultured alone
3. Circularity of co-cultured cells of uncertain origin was less than in control cancer cells cultured alone
4. Circularity of co-cultured cancer cells with some fibroblast label was less than in co-cultured cancer cells with fluorescence indistinguishable from controls
5. Circularity of co-cultured cells of uncertain origin was less than in co-cultured cancer cells with fluorescence indistinguishable from controls
6. Circularity of control fibroblasts cultured alone was less than in co-cultured fibroblasts with fluorescence indistinguishable from controls
7. Circularity of control fibroblasts cultured alone was less than in co-cultured fibroblasts with some cancer cell fluorescence
8. Circularity of control fibroblasts cultured alone was less than in co-cultured cells of uncertain origin
9. Circularity of co-cultured fibroblasts with fluorescence indistinguishable from controls was less than in co-cultured fibroblasts with some cancer cell fluorescence
10. Circularity of co-cultured fibroblasts with fluorescence indistinguishable from controls was less than in co-cultured cells of uncertain origin

Cell Line	Exp.	Statistical Significance According to Observation Number									
		1	2	3	4	5	6	7	8	9	10
SAOS-2	a	T NS	T NS	T NS	T NS	F NS	F NS		T <0.0001		T <0.0001
SAOS-2	b	T 0.0360	T 0.0030	T NS	T NS	T NS	T NS		T NS		T NS
SAOS-2	c	T <0.0001	T <0.0001	T <00001	T NS	T NS		F NS	T NS		Fil
SAOS-2	d	T 0.0170		T 0.0432		F NS	T <0.0001	T <0.0001	T <0.0001	T0.0064	T <0.0001
SAOS-2	e	F NS		F NS		T NS	T <0.0001	T <0.0001	T <0.0001	F NS	T <0.0001
SAOS-2	f	T <0.0001	T <0.0001	T <0.0001	F NS	T NS	F <0.0001	F NS	F NS	T NS	T 0.0377
SAOS-2	g	F 0.0012		T 0.0020		T <0.0001	T <0.0001	T NS	T <0.0001	F NS	T 0.009
SAOS-2	h	T NS	T 0.0411	T <0.0001	T NS	T 0.0004	F <0.0001	F 0.0128	F 0.0002	T NS	T NS
SAOS-2	i	T NS		T <0.0001		F NS	T <0.0005	F NS	T <0.0001	F 0.0035	T <0.0001
U2OS	a	F NS	T NS	T NS	T 0.0159	T NS	F NS	T NS	T <0.0001	T NS	T <0.0001
U2OS	c		T 0.0001	T <0.0001			T NS		T <0.0001		T NS
MeIRMu	a	T NS	T <0.0001	F NS	T 0.0482	F NS	F NS		T <0.0001		T <0.0001
MeiRMu	c	T NS		T NS		T NS	T NS	T NS	T 0.0024	F NS	T NS
MM200-B12	a	F 0.0003	F NS	F 0.0009	T 0.0085	F NS	F NS		T <0.0001		T <0.0001
MM200-B12	b	T NS	T 0.0026	T NS	T NS	F NS	T NS	T NS	T 0.0005	T NS	T 0.0048
MM200-B12	c		T NS	T <0.0001				F NS	T <0.0001		Fil
MM200-B12	j	T <0.0001	T 0.0033	T <0.0001	F NS	T <0.0001	T <0.0001	F NS	T 0.0070	F 0.0045	T NS
WM175	a	F 0.0005	F NS	F 0.0005	T 0.0248	T NS	F NS	T NS	T <0.0001	T NS	T <0.0001
WM175	c			T <0.0001			T NS	F NS	F NS	F NS	F NS
NM39	c			T NS				F NS	T <0.0001		Fil
Colo316	a	F NS	F NS	FXXX	T NS	T NS	T NS	T NS	T <0.0001	T NS	T<0.0001
Colo316	b	T 0.0051	T <0.0001	T <0.0001	T NS	T 0.0107	T NS	T NS	T <0.0001	T NS	T <0.0001
Colo316	c		T 0.0003	T <0.0001			T NS	F NS	T <0.0001	F NS	T NS
PEO1	c		T 0.0044	T <0.0001			F NS	T 0.0155	T <0.0001	T NS	T 0.0180

Interpretation is outlined in Table S13

Table S12. Statistical significance regarding phenotypic association between cell circularity and Exchange Units (EU), in experiments 'a' to 'j'. Observations are described as statements numbered 1 to 8, marked 'T' for 'True' or 'F' for 'False', by comparison of median values within experiments. Statistical significance is indicated in instances where this was reached at $p < 0.05$ by Mann Whitney U Test. NS indicates that the results was not statistically significant. In a number of co-cultures, some groups according to EU were absent and this accounts for 'blank' cells. Note that in experiment 'c', reversal of dye orientation necessitated multiplication of all EU values -1, to achieve direct comparability with other co-cultures.

Statements On Experimental Results

1. Circularity of co-cultured cells with 30 < EU < 50 was less than in control cancer cells cultured alone
2. Circularity of co-cultured cells with 30 < EU < 50 was less than in co-cultured cancer cells with fluorescence indistinguishable from controls (EU = 50)
3. Circularity of co-cultured cells with -10 < EU ≤ 10 was les than in control cancer cells cultured alone
4. Circularity of co-cultured cells with -10 < EU ≤ 10 was less than in co-cultured cancer cells with fluorescence indistinguishable from controls (EU = 50)
5. Circularity of control fibroblasts cultured alone was less than in co-cultured cells with -50 < EU ≤ -30
6. Circularity of co-cultured fibroblasts with fluorescence indistinguishable from controls (EU = -50) was less than in co-cultured cells with -50 < EU ≤ -30
7. Circularity of control fibroblasts cultured alone was less than in co-cultured cells with -10 < EU ≤ 10
8. Circularity of co-cultured fibroblasts with fluorescence indistinguishable from controls (EU = -50) was less than in co-cultured cells with -10 < EU ≤ 10

		Statistical Significance According to Observation Number							
Cell Line	Exp.	1	2	3	4	5	6	7	8
SAOS-2	a	T, NS	T, NS	T, NS	T, NS			T, <0.0001	T, <0.0001
SAOS-2	b	T, 0.0009	T, NS						
SAOS-2	c	T, <0.0001	T, NS	T, <0.0001	T, NS	F, NS		T, NS	
SAOS-2	d			T, 0.0122	T, NS	T, <0.0001	T, NS	T, <0.0001	T, <0.0001
SAOS-2	e	F, 0.0016	F, NS	F, NS	T, NS	T, <0.0001	T, NS	T, <0.0001	T, <0.0001
SAOS-2	f	T, <0.0001	F, 0.0390	T, <0.0001	T, 0.0235	F, NS	T, NS	F, NS	T, NS
SAOS-2	g	F, NS	T, NS	T, >0.0001	T, <0.0001	T, <0.0001	F, 0.0371	T, NS	F, 0.0085
SAOS-2	h	T, NS	T, NS	T, 0.0005	T, 0.0194	F, <0.0001	T, NS	F, 0.0033	T, NS
SAOS-2	i	T, NS	F, NS	T, <0.0001	F, NS	T, <0.0001	T, NS	T, <0.0001	T, <0.0001
U2OS	a	T, NS	T, 0.0218	T, NS	T, NS	T, NS	T, NS	T, NS	T, 0.0296
U2OS	c	T, 0.0002		T, NS				T, NS	T, NS
MeIRMu	a	T, <0.0001	T, 0.0904	F, NS	F, NS	T, NS	T, NS	T, 0.0147	T, 0.0058
MeiRMu	c	T, 0.0011	T, NS	T, 0.0014	T, NS	F, NS	F, NS	T, 0.0108	T, NS
MM200-B12	a	F, 0.0061	T, 0.0329	T, NS	T, NS	T, NS	T, NS	T, NS	T, NS
MM200-B12	b	T, 0.0069	T, NS	T, NS	F, NS	T, NS	T, NS	T, 0.0105	T, 0.0302
MM200-B12	c	T, 0.0309		T, <0.0001		T, NS		T, NS	
MM200-B12	j	T, 0.0004	T, NS			F, NS	F, 0.0015		
WM175	a	F, 0.0418	T, NS	F, NS	F, NS	T, NS	T, NS	T, 0.0027	T, 0.0022
WM175	c	T, 0.0244		T, <0.0001		F, NS	F, NS	F, NS	F, 0.0405
NM39	c			T, NS		F, NS		T, <0.0001	
Colo316	a	F, Sig	T, NS	T, NS	T, NS	T, NS	T, NS	T, 0.0158	T, 0.0031
Colo316	b	T, <0.0001	T, 0.0227	T, 0.0032	T, NS	T, 0.0007	T, 0.0021	T, 0.0111	T, 0.0100
Colo316	c	T, <0.0001		T, 0.0004		T, NS	F, NS	T, 0.0055	T, NS
PEO1	c	T, NS		T, <0.0001		T, NS	T, NS	T, <0.0001	T, 0.0067

Interpretation is outlined in Table S13

Table S13. Numerical summary of results shown in Tables S11 and S12 of the statistics relating to cell circularity in experiments 'a' to 'j'. Observations were described as statements numbered 1 to 8, and identified as 'True' or 'False' within individual experiments. For both True and False statements, distinction was made if results were statistically significant (Sig.) by Mann Whitney U Test to $p < 0.05$, or not (Not Sig.). In many co-cultures, some groups were absent so that comparison was not possible, and this reduced the number of observations possible (Obs. Poss.). To aid interpretation, the percentage of 'True statements (% Total 'True')', 'True statements that were statistically significant' (% 'True' Sig.) , and 'True statements that were not statistically significant (% 'True' Not Sig.) was calculated and is shown.

Statements On Experimental Results

Associations Between Cell Circularity and Classification According to Cartesian Plot

1. Circularity of co-cultured cancer cells with fluorescence indistinguishable from controls was less than in control cancer cells cultured alone
2. Circularity of co-cultured cancer cells with some fibroblast label was less than in control cancer cells cultured alone
3. Circularity of co-cultured cells of uncertain origin was less than in control cancer cells cultured alone
4. Circularity of co-cultured cancer cells with some fibroblast label was less than in co-cultured cancer cells with fluorescence indistinguishable from controls
5. Circularity of co-cultured cells of uncertain origin was less than in co-cultured cancer cells with fluorescence indistinguishable from controls
6. Circularity of control fibroblasts cultured alone was less than in co-cultured fibroblasts with fluorescence indistinguishable from controls
7. Circularity of control fibroblasts cultured alone was less than in co-cultured fibroblasts with some cancer cell fluorescence
8. Circularity of control fibroblasts cultured alone was less than in co-cultured cells of uncertain origin
9. Circularity of co-cultured fibroblasts with fluorescence indistinguishable from controls was less than in co-cultured fibroblasts with some cancer cell fluorescence
10. Circularity of co-cultured fibroblasts with fluorescence indistinguishable from controls was less than in co-cultured cells of uncertain origin

Associations Between Cell Circularity and Groups According to Exchange Units

11. Circularity of co-cultured cells with 30 < EU < 50 was less than in control cancer cells cultured alone
12. Circularity of co-cultured cells with 30 < EU < 50 was less than in co-cultured cancer cells with fluorescence indistinguishable from controls (EU = 50)
13. Circularity of co-cultured cells with -10 < EU ≤ 10 was les than in control cancer cells cultured alone
14. Circularity of co-cultured cells with -10 < EU ≤ 10 was less than in co-cultured cancer cells with fluorescence indistinguishable from controls (EU = 50)
15. Circularity of control fibroblasts cultured alone was less than in co-cultured cells with -50 < EU ≤ -30
16. Circularity of co-cultured fibroblasts with fluorescence indistinguishable from controls (EU = -50) was less than in co-cultured cells with -50 < EU ≤ -30
17. Circularity of control fibroblasts cultured alone was less than in co-cultured cells with -10 < EU ≤ 10
18. Circularity of co-cultured fibroblasts with fluorescence indistinguishable from controls (EU = -50) was less than in co-cultured cells with -10 < EU ≤ 10

Statement No.	True, Sig.	True, Not Sig.	False, Sig.	False, Not Sig.	Missing Observations	Total True	Obs. Poss.	% Total 'True'	% 'True' Sig.	% 'True' Not Sig.
1	6	6	3	3	6	12	18	66.7	33.3	33.3
2	11	3	0	3	7	14	17	82.4	64.7	17.6
3	13	6	3	2	0	19	24	79.2	54.2	25.0
4	4	7	0	2	11	11	13	84.6	30.8	53.8
5	4	8	0	6	6	12	18	66.7	22.2	44.4
6	5	8	2	6	3	13	21	61.9	23.8	38.1
7	3	7	1	8	5	10	19	52.6	15.8	36.8
8	19	2	1	2	0	21	24	87.5	79.2	8.3
9	1	8	2	5	8	9	16	56.3	6.3	50.0
10	14	6	0	1	3	20	21	95.2	66.7	28.6
11	12	5	4	1	2	17	22	77.3	54.5	22.7
12	4	10	1	2	7	14	17	82.4	23.5	58.8
13	12	7	0	3	2	19	22	86.4	54.5	31.8
14	3	9	0	4	8	12	16	75.0	18.8	56.3
15	5	9	1	6	3	14	21	66.7	23.8	42.9
16	1	12	2	3	6	13	18	72.2	5.6	66.7
17	13	6	1	2	2	19	22	86.4	59.1	27.3
18	11	6	2	0	5	17	19	89.5	57.9	31.6

In most co-cultures, cancer cells with some fibroblast label were less circular compared with control cancer cells alone (82.4%), or with fellow co-cultured cancer cells without detectable fibroblast labelling (84.6%), although this did not always reach statistical significance (64.7% and 30.8% respectively; Statements 2 and 4). A potential effect of uptake of cancer cell fluorescence on fibroblast circularity was less frequently seen, where fibroblasts with some cancer cell fluorescence had reduced circularity relative to both control fibroblasts and co-cultured fibroblasts indistinguishable from controls (52.6% and 56.3% of co-cultures respectively), and this was usually not statistically significant (Statements 7 and 9). Co-cultured cells in the group of uncertain origin, were often less circular compared with both control cells cultured in isolation and co-cultured cancer cells indistinguishable from controls (79.2% and 66.7% of co-cultures respectively). They were also frequently more circular compared with both control fibroblasts and co-cultured cells with fluorescence indistinguishable from control fibroblasts (87.5% and 95.2% of co-cultures respectively), and this was often statistically significant (79.2% and 66.7% respectively; Statements 3, 5, 8 and 10). Co-cultured cells with fluorescence indistinguishable from control cancer cells were less circular compared with control cancer cells cultured alone in 66.7% of co-cultures, while fibroblasts with fluorescence indistinguishable from control fibroblasts, were more circular compared with control fibroblasts in 61.9% of co-cultures. However, this was not often statistically significant and it was difficult to be convinced of a clear effect of mere co-culture alone, without detectable fluorescence transfer (Statements 1 and 6). Similar results were found considering EU, where co-cultured cells with 50 < EU ≤ 30 were often less circular compared with both control cancer cells (77.3% of co-cultures), and co-cultured cells with fluorescence indistinguishable from controls (82.4% of co-cultures). Similarly, co-cultured cells with -50 < EU ≤ -30, were often more circular compared with control fibroblasts (66.7% of co-cultures), and also compared with co-cultured cells with fluorescence indistinguishable from control fibroblasts (72.2% of co-cultures). These differences, however, were rarely statistically significant within individual co-cultures (Statements 11, 12, 15, 16). Also, in 75% of co-cultures, co-cultured cells with -10 < EU ≤ 10 had lower circularity compared with cells with EU = 50, while in 89.5% of co-cultures cells with -10 < EU ≤ 10 had greater circularity compared with co-cultured cells with EU =-50 (Statements 14 and 18).

Table S14. Statistical significance regarding phenotypic association between cell-profile area and classification of cells according to Cartesian plot group, in experiments 'a' to 'j'. Observations are described as statements numbered 1 to 10, marked 'T' for 'True' or 'F' for 'False', by comparison of median values within experiments. Statistical significance is indicated in instances where this was reached at p < 0.05 by Mann Whitney U Test. NS indicates that the results was not statistically significant. In a number of co-cultures, some groups were absent and this accounts for 'blank' cells.

Statements On Experimental Results

1. Cell-profile area of co-cultured cancer cells with fluorescence indistinguishable from controls was greater than in control cancer cells cultured alone
2. Cell-profile area of co-cultured cancer cells with some fibroblast label was greater than in control cancer cells cultured alone
3. Cell-profile area of co-cultured cells of uncertain origin was greater than in control cancer cells cultured alone
4. Cell-profile area of co-cultured cancer cells with some fibroblast label was greater than in co-cultured cancer cells with fluorescence indistinguishable from controls
5. Cell-profile area of co-cultured cells of uncertain origin was greater than in co-cultured cancer cells with fluorescence indistinguishable from controls
6. Cell-profile area of control fibroblasts cultured alone was greater than in co-cultured fibroblasts with fluorescence indistinguishable from controls
7. Cell-profile area of control fibroblasts cultured alone was greater than in co-cultured fibroblasts with some cancer cell fluorescence
8. Cell-profile area of control fibroblasts cultured alone was greater than in co-cultured cells of uncertain origin
9. Cell-profile area of co-cultured fibroblasts with fluorescence indistinguishable from controls was greater than in co-cultured fibroblasts with some cancer cell fluorescence
10. Cell-profile area of co-cultured fibroblasts with fluorescence indistinguishable from controls was greater than in co-cultured cells of uncertain origin

Cell Line	Exp.	Statistical Significance According to Observation Number									
		1	2	3	4	5	6	7	8	9	10
SAOS-2	a	T NS	T 0.0002	T NS	T NS	F NS	F NS		T 0.0032		T <0.0001
SAOS-2	b	T NS	T NS	F NS	T NS	F NS	F NS		T NS		T NS
SAOS-2	c	F NS	T NS	T 0.0037	T NS	T NS		T NS	T 0.0362		Fil
SAOS-2	d	T NS		T 0.0051		F NS	T <0.0001	T <0.0001	T <0.0001	F NS	T 0.0011
SAOS-2	e	T NS		T 0.0003		T NS	T NS	T 0.0074	T <0.0001	T NS	T <0.0001
SAOS-2	f	T NS	T NS	T NS	T NS	T NS	T NS	T NS	T 0.0002	T NS	T 0.0039
SAOS-2	g	F <0.0001		F NS		T 0.0157	T <0.0001	T NS	T <0.0001	T NS	T 0.0293
SAOS-2	h	T NS	T NS	T 0.0359	F NS	T NS	T 0.0101	T 0.0309	T <0.0001	T NS	T 0.0080
SAOS-2	i	F NS		T <0.0001		T NS	T <0.0001	T NS	T <0.0001	F 0.0001	T <0.0001
U2OS	a	T NS	T 0.0003	T 0.0013	T NS	T NS	F 0.0168	T NS	T <0.0001	T NS	T <0.0001
U2OS	c		T NS	F NS			T NS		T 0.0007		T NS
MeIRMu	a	F NS	T <0.0001	T NS	T <0.0001	T NS	T NS		T 0.0021		T 0.0020
MeiRMu	c	F NS		T <0.0001		T NS	F NS	T NS	T NS	T NS	T NS
MM200-B12	a	F NS	T NS	F NS	T NS	T NS	T NS		T 0.0007		T <0.0001
MM200-B12	b	T NS	T NS	F NS	F NS	F NS	T 0.0044	T NS	T 0.0382	T NS	T NS
MM200-B12	c		T NS	T NS				F NS	T NS		Fil
MM200-B12	j	T 0.0290	T 0.0218	T 0.0020	T NS	T 0.0166	T 0.0422	F NS	T NS	F 0.0027	F NS
WM175	a	F 0.0292	T NS	F NS	T 0.0001	T NS	T NS	T NS	T 0.0014	F NS	T 0.0009
WM175	c			T 0.0228			F NS	T NS	T NS	T 0.0227	T 0.0038
NM39	c			T 0.0076		T 0.0085		F NS	T NS		Fil
Colo316	a	F NS	T NS	T NS	F NS	F NS	F NS	T NS	T 0.0001	T NS	T <0.0001
Colo316	b	F 0.0459	T 0.0011	T NS	T NS	T NS	F NS	T NS	T 0.0008	T NS	T 0.0019
Colo316	c		T <0.0001	T 0.0026			F 0.0003	F 0.0300	T NS	T 0.0207	T 0.0010
PEO1	c		F NS	T 0.0006			F 0.0073	F NS	T 0.0001	T NS	T 0.0030

Interpretation is outlined in Table S16

Table S15. Statistical significance regarding phenotypic association between cell-profile area and Exchange Units (EU), in experiments 'a' to 'j'. Observations are described as statements numbered 1 to 8, marked 'T' for 'True' or 'F' for 'False', by comparison of median values within experiments. Statistical significance is indicated in instances where this was reached at p < 0.05 by Mann Whitney U Test. NS indicates that the results was not statistically significant. In a number of co-cultures, some groups according to EU were absent and this accounts for 'blank' cells. Note that in experiment 'c', reversal of dye orientation necessitated multiplication of all EU values -1, to achieve direct comparability with other co-cultures.

Statements On Experimental Results

1. Cell-profile area of co-cultured cells with 30 < EU < 50 was greater than in control cancer cells cultured alone
2. Cell-profile area of co-cultured cells with 30 < EU < 50 was greater than in co-cultured cancer cells with fluorescence indistinguishable from controls (EU = 50)
3. Cell-profile area of co-cultured cells with -10 < EU ≤ 10 was les than in control cancer cells cultured alone
4. Cell-profile area of co-cultured cells with -10 < EU ≤ 10 was greater than in co-cultured cancer cells with fluorescence indistinguishable from controls (EU = 50)
5. Cell-profile area of control fibroblasts cultured alone was greater than in co-cultured cells with -50 < EU ≤ -30
6. Cell-profile area of co-cultured fibroblasts with fluorescence indistinguishable from controls (EU = -50) was greater than in co-cultured cells with -50 < EU ≤ -30
7. Cell-profile area of control fibroblasts cultured alone was greater than in co-cultured cells with -10 < EU ≤ 10
8. Cell-profile area of co-cultured fibroblasts with fluorescence indistinguishable from controls (EU = -50) was greater than in co-cultured cells with -10 < EU ≤ 10

		Statistical Significance According to Observation Number							
Cell Line	Exp.	1	2	3	4	5	6	7	8
SAOS-2	a	T, 0.0028	T, NS	T, NS	F, NS			T, 0.0280	T, 0.0010
SAOS-2	b	T, NS	T, NS						
SAOS-2	c	T, 0.0225	T, NS	T, NS	T, NS	T, NS		T, NS	
SAOS-2	d			T, 0.0035	T, NS	T, <0.0001	F, 0.0040	T, <0.0001	T, 0.0407
SAOS-2	e	T, NS	T, NS	T, 0.0097	T, NS	T, 0.0024	T, 0.0230	T, 0.0001	T, 0.0007
SAOS-2	f	T, NS	F, NS	T, NS	F, NS	T, NS	T, NS	T, 0.0007	T, 0.0044
SAOS-2	g	F, <0.0001	F, NS	T, NS	T, 0.0061	T, <0.0001	F, NS	T, 0.0027	F, NS
SAOS-2	h	T, NS	F, NS	T, NS	T, NS	T, <0.0001	T, NS	T, <0.0001	T, 0.0242
SAOS-2	i	F, NS	T, NS	T, 0.0441	T, NS	T, <0.0001	F, NS	T, <0.0001	T, <0.0001
U2OS	a	T, <0.0001	T, NS	F, NS	F, NS	T, NS	T, NS	T, 0.0005	T, <0.0001
U2OS	c	F, NS		F, NS				T, NS	T, NS
MeIRMu	a	T, <0.0001	T, <0.0001	T, NS	T, NS	T, NS	T, NS	T, NS	T, NS
MeiRMu	c	T, NS	T, NS	T, <0.0001	T, NS	T, NS	T, NS	F, NS	T, NS
MM200-B12	a	T, NS	T, NS	F, NS	T, NS	T, NS	T, NS	T, NS	T, NS
MM200-B12	b	T, NS	F, NS	F, NS	F, NS	T, NS	T, NS	T, NS	T, NS
MM200-B12	c	F, NS		T, NS		F, NS		T, NS	
MM200-B12	j	T, 0.0357	T, NS			F, NS	F, 0.0009		
WM175	a	T, NS	T, 0.0003	F, NS	T, NS	T, NS	T, NS	T, 0.0211	T, 0.0209
WM175	c	F, NS		T, 0.0043		T, NS	T, 0.0167	T, NS	T, NS
NM39	c			T, NS		T, NS		T, NS	
Colo316	a	F, NS	T, NS	T, NS	T, NS	T, NS	T, NS	T, 0.0228	T, 0.0021
Colo316	b	T, 0.0020	T, NS	T, NS	T, NS	T, NS	T, NS	T, NS	T, NS
Colo316	c	T, <0.0001		T, 0.0004		F, 0.0250	T, 0.0342	F, NS	T, NS
PEO1	c	F, NS		T, NS		T, NS	T, 0.0254	T, <0.0001	T, <0.0001

Interpretation is outlined in Table S16

Table S16. Numerical summary of results shown in Tables S14 and S15 of the statistics relating to cell-profile area in experiments 'a' to 'j'. Observations were described as statements numbered 1 to 8, and identified as 'True' or 'False' within individual experiments. For both True and False statements, distinction was made if results were statistically significant (Sig.) by Mann Whitney U Test to $p < 0.05$, or not (Not Sig.). In many co-cultures, some groups were absent so that comparison was not possible, and this reduced the number of observations possible (Obs. Poss.). To aid interpretation, the percentage of 'True statements (% Total 'True')', 'True statements that were statistically significant' (% 'True' Sig.) , and 'True statements that were not statistically significant (% 'True' Not Sig.) was calculated and is shown.

Statements On Experimental Results

Associations Between Cell-Profile Area and Classification According to Cartesian Plot

1. Cell-profile area of co-cultured cancer cells with fluorescence indistinguishable from controls was greater than in control cancer cells cultured alone
2. Cell-profile area of co-cultured cancer cells with some fibroblast label was greater than in control cancer cells cultured alone
3. Cell-profile area of co-cultured cells of uncertain origin was greater than in control cancer cells cultured alone
4. Cell-profile area of co-cultured cancer cells with some fibroblast label was greater than in co-cultured cancer cells with fluorescence indistinguishable from controls
5. Cell-profile area of co-cultured cells of uncertain origin was greater than in co-cultured cancer cells with fluorescence indistinguishable from controls
6. Cell-profile area of control fibroblasts cultured alone was greater than in co-cultured fibroblasts with fluorescence indistinguishable from controls
7. Cell-profile area of control fibroblasts cultured alone was greater than in co-cultured fibroblasts with some cancer cell fluorescence
8. Cell-profile area of control fibroblasts cultured alone was greater than in co-cultured cells of uncertain origin
9. Cell-profile area of co-cultured fibroblasts with fluorescence indistinguishable from controls was greater than in co-cultured fibroblasts with some cancer cell fluorescence
10. Cell-profile area of co-cultured fibroblasts with fluorescence indistinguishable from controls was greater than in co-cultured cells of uncertain origin

Associations Between Cell Profile Area and Groups According to Exchange Units

11. Cell-profile area of co-cultured cells with $30 < EU < 50$ was greater than in control cancer cells cultured alone
12. Cell-profile area of co-cultured cells with $30 < EU < 50$ was greater than in co-cultured cancer cells with fluorescence indistinguishable from controls ($EU = 50$)
13. Cell-profile area of co-cultured cells with $-10 < EU \leq 10$ was les than in control cancer cells cultured alone
14. Cell-profile area of co-cultured cells with $-10 < EU \leq 10$ was greater than in co-cultured cancer cells with fluorescence indistinguishable from controls ($EU = 50$)
15. Cell-profile area of control fibroblasts cultured alone was greater than in co-cultured cells with $-50 < EU \leq -30$
16. Circularity of co-cultured fibroblasts with fluorescence indistinguishable from controls ($EU = -50$) was greater than in co-cultured cells with $-50 < EU \leq -30$
17. Cell-profile area of control fibroblasts cultured alone was greater than in co-cultured cells with $-10 < EU \leq 10$
18. Cell-profile area of co-cultured fibroblasts with fluorescence indistinguishable from controls ($EU = -50$) was greater than in co-cultured cells with $-10 < EU \leq 10$

Statement No.	True, Sig.	True, Not Sig.	False, Sig.	False, Not Sig.	Missing Observations	Total True	Obs. Poss.	% Total 'True'	% 'True' Sig.	% 'True' Not Sig.
1	1	8	3	6	6	9	18	50.0	5.6	44.4
2	6	10	0	1	7	16	17	94.1	35.3	58.8
3	12	6	0	6	0	18	24	75.0	50.0	25.0
4	2	8	0	3	11	10	13	76.9	15.4	61.5
5	3	11	0	5	5	14	19	73.7	15.8	57.9
6	6	6	3	6	3	12	21	57.1	28.6	28.6
7	3	11	1	4	5	14	19	73.7	15.8	57.9
8	17	7	0	0	0	24	24	100.0	70.8	29.2
9	2	10	2	2	8	12	16	75.0	12.5	62.5
10	16	4	0	1	3	20	21	95.2	76.2	19.0
11	7	8	1	6	2	15	22	68.2	31.8	36.4
12	2	11	0	4	7	13	17	76.5	11.8	64.7
13	6	11	0	5	2	17	22	77.3	27.3	50.0
14	1	11	0	4	8	12	16	75.0	6.3	68.8
15	5	13	1	2	3	18	21	85.7	23.8	61.9
16	4	10	2	2	6	14	18	77.8	22.2	55.6
17	11	9	0	2	2	20	22	90.9	50.0	40.9
18	10	8	0	1	5	18	19	94.7	52.6	42.1

In most co-cultures, cancer cells with some fibroblast label had greater cell-profile area compared with control cancer cells alone (94.1%), or with fellow co-cultured cancer cells without detectable fibroblast labelling (76.9%), although this usually failed to reach statistical significance (35.3% and 15.4% respectively; Statements 2 and 4). Uptake of cancer cell fluorescence was associated with increased fibroblast cell-profile area, such that fibroblasts with some cancer cell fluorescence had reduced cell-profile area relative to control fibroblasts and co-cultured fibroblasts indistinguishable from controls in 73.7% and 75% of co-cultures respectively, but this was usually not statistically significant (Statements 7 and 9). Co-cultured cells in the group of uncertain origin, frequently had greater cell-profile area compared with control cultured cells and co-cultured cancer cells indistinguishable from controls (75% and 73.7% of co-cultures respectively), while they also had lower cell-profile area compared with control fibroblasts and co-cultured fibroblasts with fluorescence indistinguishable from controls in the great majority of co-cultures (100% and 95.2% of co-cultures respectively), and this was frequently statistically significant (70.8% and 76.2% respectively; Statements 3, 5, 8 and 10). There seemed to be no consistent difference in cell-profile area of co-cultured cells with fluorescence indistinguishable from controls, relative to controls (Statements 1 and 6). Similar results were found considering EU, where co-cultured cells with $50 < EU \leq 30$ often had greater cell-profile area compared with control cancer cells (68.2% of co-cultures), and co-cultured cells with fluorescence indistinguishable from controls (76.5% of co-cultures). Co-cultured cells with $-50 < EU \leq -30$, frequently had lower cell profile area compared with control fibroblasts (85.7% of co-cultures) as well as with co-cultured cells indistinguishable in fluorescence from control fibroblasts (77.8% of co-cultures), but these differences were not often statistically significant (Statements 11, 12, 15, 16). Also, in 75% of co-cultures, co-cultured cells with $-10 < EU \leq 10$ had greater cell-profile area compared with co-cultured cancer cells with fluorescence indistinguishable from control cancer cells; while in 94.7% of co-cultures, cells in the $-10 < EU \leq 10$ group had lower cell-profile area compared with cells with $EU = -50$ (Statements 14 and 18).

Table S17. Median values for cell-profile area in control cells cultured in isolation, as well as in co-cultured cells according to Cartesian Plot grouping in each experiment for assessment of global DNA methylation (experiments 'm' to 'p'). The experiment 'n' marked * is that shown in Figure S12. Median values across all experiments are shown.

Experiment	Median Cell-Profile Area (mm^2)						
	Control SAOS-2	Cells Indistinguishable From SAOS-2 Controls	SAOS-2 With Some Fibroblast Labelling	Cells of Uncertain Origin	Fibroblasts With Some SAOS-2 Labelling	Cells Indistinguishable From Fibroblast Controls	Control Fibroblasts
m	1,498	2,038	2,356	2,686		2,388	2,966
* n	953	926	1,058	962	1,376	1,060	2,429
o	531	624		982	1,084	1,032	1,392
p	540	691		1,258		896	1,491
Median	747	808	1,707	1,120	1,230	1,046	1,960

Control SAOS-2 cultured in isolation had lower cell-profile area compared with control human dermal fibroblasts ($p < 0.006$, Paired T Test considering all experiments together; $p < 0.0001$, Mann Whitney U Test for each experiment individually). This difference was maintained when co-cultured cells with fluorescence indistinguishable from that in controls were considered ($p < 0.025$, Paired T Test considering all co-cultures together; $p < 0.0001$ in experiment 'o', $p = 0.0023$ in experiment 'p', not statistically significant for experiments 'm' and 'n' by Mann Whitney U Test in individual experiments). Notwithstanding irregularity of uncommonly high values for the group 'SAOS-2 with some fibroblast labelling', the general pattern of data across groups was similar to that observed for experiments 'a' to 'j'.

Table S18. Median values for cell circularity in control cells cultured in isolation, as well as in co-cultured cells according to Cartesian Plot grouping in each experiment for assessment of global DNA methylation (experiments 'm' to 'p'). The experiment 'n' marked * is that shown in Figure S12. Median values across all experiments are shown.

	Median Cell Circularity						
Experiment	Control SAOS-2	Cells Indistinguishable From SAOS-2 Controls	SAOS-2 With Some Fibroblast Labelling	Cells of Uncertain Origin	Fibroblasts With Some SAOS-2 Labelling	Cells Indistinguishable From Fibroblast Controls	Control Fibroblasts
m	0.48	0.40	0.36	0.44		0.41	0.45
* n	0.64	0.64	0.61	0.55	0.54	0.56	0.39
o	0.69	0.69		0.54	0.52	0.53	0.45
p	0.64	0.61		0.48		0.50	0.49
Median	0.64	0.62	0.49	0.51	0.53	0.52	0.45

Control SAOS-2 cultured in isolation had higher cell circularity compared with control human dermal fibroblasts ($p < 0.05$, Paired T Test considering all experiments together; $p < 0.0001$ for experiments 'n', 'o' and 'p', and not statistically significant for experiment 'm', Mann Whitney U Test for each experiment individually). This difference was maintained when co-cultured cells with fluorescence indistinguishable from that in controls were considered, but did not reach statistical significance considering all experiments together (Paired T Test), or in experiments 'm' and 'n' considered alone (Mann Whitney U Test), although statistical significance was reached in experiments 'o' and 'p' ($p < 0.0001$, Mann Whitney U Test). The general pattern of data across EU groups was similar to that observed for experiments 'a' to 'j'.

Table S19. Median values for cell-profile area in control cells cultured in isolation, as well as in co-cultured cells according to EU grouping in each experiment for assessment of global DNA methylation (experiments 'm' to 'p'). The experiment 'n' marked * is that shown in Figure S12. Median values across all experiments are shown.

	Median Cell-Profile Area (mm^2)								
Experiment	Control SAOS-2	EU=50	30<EU≤50	10<EU≤30	-10<EU≤10	-30<EU≤-10	-50<EU≤-30	EU = -50	Control Fibroblasts
m	1,498	2,038	2,751	2,096	2,558	2,334	2,908	2,388	2,966
* n	953	926	1,014	925	1,202	1,063	1,446	1,060	2,429
o	531	624		722	666	1,152	1,193	1,032	1,392
p	540	691	1,329	832	1,254	1,357	1,429	896	1,491
Median	747	808	1,329	879	1,228	1,254	1,438	1,046	1,960

Control SAOS-2 cultured in isolation had lower cell-profile area compared with control human dermal fibroblasts ($p < 0.006$, Paired T Test considering all experiments together; $p < 0.0001$, Mann Whitney U Test for each experiment individually). This difference was maintained when co-cultured cells with fluorescence indistinguishable from that in controls were considered ($p < 0.025$, Paired T Test considering all experiments together; $p < 0.0001$ in experiment 'o', $p = 0.0023$ in experiment 'p', not statistically significant for experiments 'm' and 'n' by Mann Whitney U Test in individual experiments). Notwithstanding irregularity of uncommonly high values for the group $30 < EU \leq 50$, the general pattern of data was similar to that observed in co-cultures 'a' to 'j'.

Table S20. Median values for cell circularity in control cells cultured in isolation, as well as in co-cultured cells according to EU grouping in each experiment for assessment of global DNA methylation (experiments 'm' to 'p'). The experiment 'n' marked * is that shown in Figure S12. Median values across all experiments are shown.

	Median Cell Circularity								
Experiment	Control SAOS-2	EU=50	30<EU≤50	10<EU≤30	-10<EU≤10	-30<EU≤-10	-50<EU≤-30	EU = -50	Control Fibroblasts
m	0.48	0.40	0.39	0.43	0.48	0.46	0.43	0.41	0.45
* n	0.64	0.64	0.60	0.57	0.49	0.56	0.51	0.56	0.39
o	0.69	0.69		0.74	0.76	0.46	0.51	0.53	0.45
p	0.64	0.61	0.50	0.51	0.51	0.43	0.40	0.50	0.49
Median	0.64	0.62	0.50	0.54	0.50	0.46	0.47	0.52	0.45

Control SAOS-2 cultured in isolation had higher cell circularity compared with control human dermal fibroblasts ($p < 0.05$, Paired T Test considering all experiments together; $p < 0.0001$ for experiments 'n', 'o' and 'p', and not statistically significant for experiment 'm', Mann Whitney U Test for each experiment individually). This difference was maintained when co-cultured cells with fluorescence indistinguishable from that in controls were considered, but did not reach statistical significance considering all experiments together (Paired T Test), or in experiments 'm' and 'n' considered alone (Mann Whitney U Test), although statistical significance was reached in experiments 'o' and 'p' ($p < 0.0001$, Mann Whitney U Test). The general pattern of data across EU groups was similar to that observed in experiments 'a' to 'j'.

Table S21. Median values for normalized 5mc in control cells cultured in isolation, as well as in co-cultured cells according to Cartesian Plot grouping, in each experiment for assessment of global DNA methylation (experiments 'm' to 'p'). The experiment 'p' marked * is that shown in Figure 6 in the main manuscript. Median values across all experiments are shown.

	Median Cell-Profile Area (mm^2)						
Experiment	Control SAOS-2	Cells Indistinguishable From SAOS-2 Controls	SAOS-2 With Some Fibroblast Labelling	Cells of Uncertain Origin	Fibroblasts With Some SAOS-2 Labelling	Cells Indistinguishable From Fibroblast Controls	Control Fibroblasts
m	71.9	1.5	2.4	6.9		2.0	101.3
n	21.4	16.0	11.5	6.1	9.3	9.8	100.0
o	29.6	51.7		54.0	57.0	75.0	100.0
* p	14.9	20.3		9.2		20.4	100.0
Median	25.5	18.2	6.9	8.0	33.2	15.1	100.0

Control human dermal fibroblasts cultured in isolation had appreciably higher normalized 5mc compared with control SAOS-2 ($p < 0.015$ Paired T Test considering all experiments together; $p < 0.015$ for experiment 'm', and $p < 0.0001$ for experiments 'n', 'o', and 'p', Mann Whitney U Test). Similarly, control fibroblasts had higher normalized 5mc compared with co-cultured cells with DiD-DiO fluorescence indistinguishable from control fibroblasts ($p < 0.022$, Paired T Test considering all experiments together; $p < 0.0001$ for experiments 'm', 'n', and 'p', and $p < 0.0025$ for experiment 'o', Mann Whitney U Test). There was no clear relationship between normalized 5mc in control SAOS-2 cultured alone, and that in co-cultured cells grouped according to Cartesian Plot.

Table S22. Median values for normalized 5mc in control cells cultured in isolation, as well as in co-cultured cells according to EU grouping, in each experiment for assessment of global DNA methylation (experiments 'm' to 'p'). The experiment 'p' marked * is that shown in Figure 6 in the main manuscript. Median values across all experiments are shown.

	Median Cell Circularity								
Experiment	Control SAOS-2	EU=50	30<EU≤50	10<EU≤30	-10<EU≤10	-30<EU≤-10	-50<EU≤-30	EU = -50	Control Fibroblasts
m	71.9	1.5	6.1	7.9	6.3	3.5	7.1	2.0	101.3
n	21.4	16.0	9.7	7.6	10.3	1.1	9.2	9.8	100.0
o	29.6	51.7		54.8	52.8	47.2	54.5	75.0	100.0
* p	14.9	20.3	7.6	11.8	13.2	18.6	7.3	20.4	100.0
Median	25.5	18.2	7.6	9.9	11.7	11.0	8.3	15.1	100.0

Control human dermal fibroblasts cultured in isolation had appreciably higher normalized 5mc compared with control SAOS-2 ($p < 0.015$ Paired T Test considering all experiments together; $p < 0.015$ for experiment 'm', and $p < 0.0001$ for experiments 'n', 'o', and 'p', Mann Whitney U Test). Similarly, control fibroblasts had higher normalized 5mc compared with co-cultured cells with fluorescence indistinguishable from control fibroblasts ($p < 0.022$, Paired T Test considering all experiments together; $p < 0.0001$ for experiments 'm', 'n', and 'p', and $p < 0.0025$ for experiment 'o', Mann Whitney U Test). There was no clear relationship between normalized 5mc in control SAOS-2 cultured alone, and that in co-cultured cells grouped according to EU.

MATLAB Script For Cartesian Plot Analysis

Pages

36-80 Annotated MATLAB script

Explanatory annotations are in 'Green' .

The script provided can be directly copied into MATLAB and used as detailed below.

```
% SCRIPT FOR CARTESIAN PLOT ANALYSIS
%-------------------------------------------------------------

% PURPOSE OF THE SCRIPT
%   This is a script for Cartesian Plot analysis of inter-cellular
% fluorescence transfer between two cell types, each labelled with one
% of two separate fluorescent labels.
%   The analysis defines transfer in terms of 'Exchange Units', and also
%   identifies cells as belonging to one of five separate groups relative
%   to controls.
%   Exchange units and group identity for each cell can then be related to
%   other phenotypic features, such as cell surface profile area or
%   circularity as in the current script, or other parameters if wished.
%   The script is written assuming use of Red and Green fluorescence,
% but any fluorescent marker can substitute for either of these.

% CONCEPTUAL OVERVIEW
%    Control green cells are ultimately aligned along an X axis, and
% control red cells are aligned along a Y axis, according to the
% level of fluorescence of each individual cell.
%    Cells in co-cultures where transfer between each cell type can occur,
% are marked as dots scattered in the Cartesian plane, according to
% their individual green and red fluorescence relative to controls.

% Cells are then classified as belonging to one of five groups:
%       Group 1: Cells with fluorescence indistinguishable from Control
%               Red Cells, being cells that have red fluorescence in the
%               range of control red cells, and green fluorescence in
%               the range of background for control red cells
%       Group 2: Red cells with some green fluorescence, being cells that
%               have red fluorescence in the range of control red cells,
%               and green fluorescence less than that of control green
%               cells
%       Group 3: Cells of uncertain origin, being cells that have red and
%               green fluorescence that overlaps with both red and green
%               control cell populations
%       Group 4: Green cells with some red fluorescence, being cells that
%               have green fluorescence in the range of green control
%               cells, and red fluorescence less than that in red control
%               cells.
%       Group 5: Cells with fluorescence indistinguishable from Control
%               Green Cells, being cells that have red fluorescence in the
%               range of control Green cells, and red fluorescence in
%               the range of background for control green cells
% Cells are also assigned a single 'Exchange Unit' value
%    If there were complete mixing of all fluorescence between opposite
% cell types, cells would cluster about a bisecting angle of 45o
%    Divergence from the 45o bisecting angle, thus provides a measure of
% the extent to which cells in co-cultures are similar in fluorescence
% relative to controls, and also of transfer
%    Note that highly variable fluorescence in starting populations, makes
% direct fluorescence measurements less reliable compared with this
% angular measure.
%    For convenience, cells on the bisecting line are assigned Exchange
```

```
% values of 0, those on the Y axis are assigned -50 Exchange Units, and
% those on the X axis are assigned negative 50 Exchange Units.

% ALGORITHMIC APPROACH
%    Background red and green values determined from controls, are
% subtracted from input red and green values.
%   Control fluorescence is normalized relative to an arbitrary
% value of '100', to permit ready comparison between co-cultures.
% The Median value of either red or green controls, is defined as '100'.
%   Outliers with negligible fluorescence, or outrageously high
% fluorescence, are removed from controls and the co-cultures
%   Red and Green fluorescence is normalized relative to '100'
%   In the current script, pixel values for area are converted to
% square micrometers but this may not be required in all applications.
%   Cells in co-cultures that are indistinguishable from controls are
% identified and assigned Exchange Unit values accordingly.
%   Exchange Units for remaining cells in co-cultures are determined for
% each cell plotted on the plane, by calculation of the angle subtended
% to the origin, and subtraction relative to the bisecting line
%   Cells in co-cultures are identified as belonging to one of five groups:
%    as defined above.
%   Results are plotted on the cartesian plane
%   In this script Exchange Units are related to circularity and
% Cell Profile Surface Area, and plotted accordingly, although other
% parameters can be studied by substitution using this approach if wished.

%-----------------------------------------------------------------
%-----------------------------------------------------------------

% INPUT DATA AND FILES
% Three files are required for the script to run, one for each of the
% controls and one for the co-culture, and these are loaded as numeric
% matrices.
% NOTE: All three input data files accord to the below column order:
%Column Number   Contents
% 1          Cell Identification Number
% 2          Image Number
% 3          Surface Area (in pixels)
% 4          Perimeter (in pixels)
% 5          Circularity
% 6          Total Green in segmented area
% 7          Total Red in segmented area


% The Operator using this script must either use the below indicated file
% names, or correct the script to use an appropriate file name.

% CONTROL GREEN Cells-
   GreenCont = GreenControlFileName;

% CONTROL RED cells-
   RedCont = RedControlFileName;

% CO-CULTURE Cells-
```

```
    CoCult = CoCultureFileName;

% The Correct Number of Square Micrometers per Pixel must be inserted for
% the variable pxl
 pxl = 0.162;

% The Maximum Number of Cells in a Group in Co-culture is cMAX, and must be
% recorded. In the current script, it is given as 1000.
cMAX = 1000;

% Graphical output is sensitive to ranges of data, and it is convenient to
% for values adjusting the ranges of graphs to be in this part of the
% script. The Operator must adjust the below given values to suit data from
% individual co-cultures.

% Values Convenient for Preparation of the Linear Scattergram
% Convenient x value for Red Controls in Linear Scattergram
% Convenient upper bound for x axis
xlinhi = 120;
% Convenient upper bound for y axis
ylinhi = 120;

% Values Convenient for Preparation of the Log Scattergram
% Convenient x value for Red Controls in Log Scattergram
lgx = 0.4;
% Conveneint y value for Green Controls in Log Scattergram
lgy = 10;
% Convenient lower bound for x axis
xlglow = 0.4;
% Convenient upper bound for x axis
xlghi = 120;
% Convenient lower bound for y axis
ylglow = 10;
% Convenient upper bound for y axis
ylghi = 120;


% Convenient upper bound for Area in Area Plot
yAr = 4500;

%-------------------------------------------------------------------
% LIST OF OUTPUTS FROM SCRIPT
% The script generates a series of matrices with results that are helpful.
% These results are saved in whichever directory the Operator elects to
% use. The saved files and location of data in columns is given as below.

% Graphical output
   % Cartesian plot of all cells color coded as to group on a linear scale
   % Cartesian plot of all cells color coded as to group on a log scale
   % Plot of cell circularity all cells color coded as to group and
   %    displayed according to Exchange Units, showing median values
   %    across bands of 20 Exchange Units.
   % Plot of cell-profile area all cells color coded as to group and
   %    displayed according to Exchange Units, showing median values
```

```
  %    across bands of 20 Exchange Units.

% AreaEUData.txt gives area of all cells in square micrometers in grouped
% Exchange Units
  % Column 1: Control Red Cells
  % Column 2: Group 1
  % Column 3: Neg50toNeg30
  % Column 4: Neg30toNeg10
  % Column 5: Neg10toPos10
  % Column 6: Pos10toPos30
  % Column 7: Pos30toPos50
  % Column 8: Group 5
  % Column 9: Control Green Cells

% AreaGroupData.txt gives area for all cells in square micrometers
% according to Cartesian plot group
  % Column 1: Control Red Cells
  % Column 2: Group 1
  % Column 3: Group 2
  % Column 4: Group 3
  % Column 5: Group 4
  % Column 6: Group 5
  % Column 7: Control Green Cells

% CircEUData.txt gives the circularity of all cells in grouped Exchange
% Units
  % Column 1: Control Red Cells
  % Column 2: Group 1
  % Column 3: Neg50toNeg30
  % Column 4: Neg30toNeg10
  % Column 5: Neg10toPos10
  % Column 6: Pos10toPos30
  % Column 7: Pos30toPos50
  % Column 8: Group 5
  % Column 9: Control Green Cells

% CircGroupData.txt gives the circularity of all cells according to
% Cartesian plot group
  % Column 1: Control Red Cells
  % Column 2: Group 1
  % Column 3: Group 2
  % Column 4: Group 3
  % Column 5: Group 4
  % Column 6: Group 5
  % Column 7: Control Green Cells

% CoCultureData.txt gives all data for co-cultures after processing
% Column 1  Corrected Red (by Subtration of Av red from Green Controls)
% Column 2  Corrected Green (by Subtraction of Av green from Red Controls)
% Column 3  Circularity
% Column 4  Area in micrometers
% Column 5  Red Value Relative to Median = 100
% Column 6  Green Value Relative to Median = 100
```

% GreenControlData.txt gives all data for green controls after processing
% Column 1 Corrected Red (by Subtration of Av red from Green Controls)
% Column 2 Corrected Green (by Subtraction of Av green from Red Controls)
% Column 3 Circularity
% Column 4 Area in micrometers
% Column 5 Red Value Relative to Median = 100
% Column 6 Green Value Relative to Median = 100

% MedianEUArea.txt gives the median area in square micrometers in grouped
% Exchange Units.
% Column 1: Control Red Cells
% Column 2: Group 1
% Column 3: Neg50toNeg30
% Column 4: Neg30toNeg10
% Column 5: Neg10toPos10
% Column 6: Pos10toPos30
% Column 7: Pos30toPos50
% Column 8: Group 5
% Column 9: Control Green Cells

% MedianEUCircularity.txt gives the median circularity in square
% micrometers in grouped Exchange Units
% Column 1: Control Red Cells
% Column 2: Group 1
% Column 3: Neg50toNeg30
% Column 4: Neg30toNeg10
% Column 5: Neg10toPos10
% Column 6: Pos10toPos30
% Column 7: Pos30toPos50
% Column 8: Group 5
% Column 9: Control Green Cells

% MedianGroupArea.txt gives the median area in square micrometers according
% to Cartesian plot group
% Column 1: Control Red Cells
% Column 2: Group 1
% Column 3: Group 2
% Column 4: Group 3
% Column 5: Group 4
% Column 6: Group 5
% Column 7: Control Green Cells

% MedianGroupCircularity.txt gives the median circularity according to
% Cartesian plot group
% Column 1: Control Red Cells
% Column 2: Group 1
% Column 3: Group 2
% Column 4: Group 3
% Column 5: Group 4
% Column 6: Group 5
% Column 7: Control Green Cells

% RedControlData.txt gives all data for green controls after processing
% Column 1 Corrected Red (by Subtration of Av red from Green Controls)

```
% Column 2  Corrected Green (by Subtraction of Av green from Red Controls)
% Column 3  Circularity
% Column 4  Area in micrometers
% Column 5  Red Value Relative to Median = 100
% Column 6  Green Value Relative to Median = 100

%-------------------------------------------------------------------
% START OF ANALYTICAL SCRIPT
%-------------------------------------------------------------------
% DETERMINATION OF FLUORESCENCE BACKGROUND FROM CONTROLS
% RBkg = Red background by summation of Red from Green Controls,
% and division by the sum of areas from Green Controls
FlTot = sum(GreenCont(:,7));
ArTot = sum(GreenCont(:,3))*pxl;
RBkg = FlTot/ArTot;

% GBkg = Green background by summation of Green from Red Controls,
% and division by the sum of areas from Red Controls
FlTot = sum(RedCont(:,6));
ArTot = sum(RedCont(:,3))*pxl;
GBkg = FlTot/ArTot;

clear FlTot ArTot

%-------------------------------------------------------------------

% CREATION OF TEMPORARY MATRICES FOR CONTROL FLUORESCENCE INTENSITIES
% AND AREAS, AND PREPARING TO LOAD DATA INTO ANALYSIS MATRICES

% GreenInt AND RedInt - Column 1 = Fluorescence, Column 2 = Area
gsz = size(GreenCont);
g = gsz(1,1);
GreenInt = zeros(g,2);

rsz = size(RedCont);
r = rsz(1,1);
RedInt = zeros(r,2);

% Calculation of Areas in Square Micrometers and Loading Into Matrices
GreenInt (:,2) = GreenCont(:,3)*pxl;
RedInt (:,2) = RedCont(:,3)*pxl;

% Loading fluorescence intensities by (Fluorescence / Area) - Background
for i = 1:g
   GreenInt(i,1) = (GreenCont(i,6)/GreenInt(i,2))-GBkg;
end

for i = 1:r
   RedInt(i,1) = (RedCont(i,7)/RedInt(i,2))-RBkg;
end

% % IDENTIFYING FLUOROESCENCE OUTLIERS IN TEMPORARY MATRICES
% Matrix for sorting Green values = Srt
Srt = GreenInt(:,1);
```

```
% Immediately Below Script for identification Quartiles by:
%Author: David Ferreira - Federal University of Amazonas
%Contact: ferreirad08@gmail.com
% Yielding Q [quartile 1, quartile 2, quartile 3]
% and Interquartile values IQR (= Quartile 3 - Quartile 1)
X = sort(Srt);
if isrow(X), X = X'; end
[n,m] = size(X);
if isscalar(X), X(2,1) = 0; end
LMU = [(n+3)/4; (n+1)/2; (3*n+1)/4];
i = floor(LMU);
d = LMU-i;
Q = X(i,:) + (X(i+1,:)-X(i,:)).*repmat(d,1,m);
IQR = Q(3,:)-Q(1,:);
if isvector(X), Q = Q'; end

% Calculating Outlier Values (LowVal, HighVal) by the formula:
% Low Outliers are < than.. LowVal = Q1 - (1.5*IQR)
% High Outliers are > than .. HighVal = Q3 + (1.5*IQR)
Q1 = Q(1,1);
Q2 = Q(1,3);
LowVal = Q1 - (1.5*IQR);
HighVal = Q2 + (1.5*IQR);

% Substituting outlier Green fluorescence values with 0
for i = 1:g
   if GreenInt(i,1)<LowVal||GreenInt(i,1)>HighVal||GreenInt(i,1)<= 0
      GreenInt(i,1) = 0;
      GreenInt(i,2) = 0;
   end
end


% Matrix for sorting Red values = Srt
Srt = RedInt(:,1);
% Immediately Below Script for identification Quartiles by:
%Author: David Ferreira - Federal University of Amazonas
%Contact: ferreirad08@gmail.com
% Yielding Q [quartile 1, quartile 2, quartile 3]
% and Interquartile values IQR (= Quartile 3 - Quartile 1)
X = sort(Srt);
if isrow(X), X = X'; end
[n,m] = size(X);
if isscalar(X), X(2,1) = 0; end
LMU = [(n+3)/4; (n+1)/2; (3*n+1)/4];
i = floor(LMU);
d = LMU-i;
Q = X(i,:) + (X(i+1,:)-X(i,:)).*repmat(d,1,m);
IQR = Q(3,:)-Q(1,:);
if isvector(X), Q = Q'; end

% Calculating Outlier Values (LowVal, HighVal) by the formula:
% Low Outliers are < than.. LowVal = Q1 - (1.5*IQR)
% High Outliers are > than .. HighVal = Q3 + (1.5*IQR)
```

```
Q1 = Q(1,1);
Q2 = Q(1,3);
LowVal = Q1 - (1.5*IQR);
HighVal = Q2 + (1.5*IQR);

% Substituting outlier Red fluorescence values with 0
for i = 1:r
   if RedInt(i,1) < LowVal || RedInt (i,1) > HighVal || RedInt (i,1) <= 0
      RedInt(i,1) = 0;
      RedInt(i,2) = 0;
   end
end


% Counting the number of control cells remaining (GrCells, RdCells)
counter = 0;
for i = 1:g
   if GreenInt(i,1) > 0
      counter = counter + 1;
   else
      continue
   end
end
GrCells = counter;

counter = 0;
for i = 1:r
   if RedInt(i,1) > 0
      counter = counter + 1;
   else
      continue
   end
end
RdCells = counter;

% Clearing values no longer needed
clear counter gsz HiGren HiRed i LowGren LowRed MedGren MedRed rsz
clear d GrCt HighVal IQR LMU LowVal  m  nQ Q1 Q2 Srt X n Q
%--------------------------------------------------------------------

% CREATING ANALYSIS MATRICES AND LOADING DATA INTO THESE MATRICES

% CREATION OF MATRICES
% GrCt is FOR GREEN CONTROL - ULTIMATE CONTENTS WILL BE:
% Row Number = number of non-outliers in Green Control
% Column 1  Corrected Red (by Subtration of Av red from Green Controls)
% Column 2  Corrected Green (by Subtraction of Av green from Red Controls)
% Column 3  Circularity
% Column 4  Area in micrometers
% Column 5  Red Value Relative to Median = 100
% Column 6  Green Value Relative to Median = 100

% RdCt is FOR RED CONTROL - ULTIMATE CONTENTS WILL BE:
% Column 1  Corrected Red (by Subtraction of Av red from Green Controls)
```

```
% Column 2  Corrected Green (by Subtraction of Av green from Red Controls)
% Column 3  Circularity
% Column 4  Area in micrometers
% Column 5  Red Value Relative to Median = 100
% Column 6  Green Value Relative to Median = 100

% CoClt is FOR CO-CULTURE MATRIX ULTIMATE CONTENTS WILL BE:
% Column 1  Corrected Red (by Subtraction of Av red from Green Controls)
% Column 2  Corrected Green (by Subtraction of Av green from Red Controls)
% Column 3  Circularity
% Column 4  Area in micrometers
% Column 5  Red Value Relative to Median = 100
% Column 6  Green Value Relative to Median = 100
% Column 7  Anti-Tan in radians
% Column 8  Exchange Units
% Column 9  Group Where:
%              Group 1 = Indistinguishable from Control Red
%              Group 2 = Red cells with some Green
%              Group 3 = Cells of uncertain origin
%              Group 4 = Green cells with some Red
%              Group 5 = Indistinguishable from Control Green


GrCt = zeros(GrCells,6);
RdCt = zeros(RdCells,6);

% Determininig the number of rows for CoClt = rn and clearing  s & rn
% and then creating CoClt
s = size(CoCult);
cc = s(1,1);
CoClt = zeros(cc,9);

% LOADING DATA INTO GrCt
counter = 1;

for i=1:g
   if GreenInt(i,1) > 0
      % Area
      GrCt(counter,4) = GreenCont(i,3)*pxl;
      % Circularity
      GrCt(counter,3) = GreenCont(i,5);
      % Red
      GrCt(counter,1) = GreenCont(i,7)/GrCt(counter,4);
      % Green
      GrCt(counter,2) = GreenCont(i,6)/GrCt(counter,4);
      counter = counter + 1;
   else
      continue
   end
end


% LOADING DATA INTO RdCt
counter = 1;
```

```
for i=1:r
  if RedInt(i,1) > 0
    % Area
    RdCt(counter,4) = RedCont(i,3)*pxl;
    % Circularity
    RdCt(counter,3) = RedCont(i,5);
    % Red
    RdCt(counter,1) = RedCont(i,7)/RdCt(counter,4);
    % Green
    RdCt(counter,2) = RedCont(i,6)/RdCt(counter,4);
    counter = counter + 1;
  else
    continue
  end
end

% LOADING DATA INTO CoClt
for i = 1:cc
  % Area
  CoClt(i,4) = CoCult(i,3)*pxl;
  % Circularity
  CoClt(i,3) = CoCult(i,5);
  % Red
  CoClt(i,1) = CoCult(i,7)/CoClt(i,4);
  % Green
  CoClt(i,2) = CoCult(i,6)/CoClt(i,4);
end

clear CoCult counter g GreenCont GreenInt i pxl r RedCont RedInt s
clear RdCells GrCells


%------------------------------------------------------------------------
% SUBTRACTING BACKGROUND FROM RED AND GREEN VALUES

% Subracting RBkg and GBkg from red and green intensity values in
% GrCt, RdCt and CoClt

GrCt(:,1) = GrCt(:,1)-RBkg;
GrCt(:,2) = GrCt(:,2)-GBkg;
RdCt(:,1) = RdCt(:,1)-RBkg;
RdCt(:,2) = RdCt(:,2)-GBkg;
CoClt(:,1) = CoClt(:,1)-RBkg;
CoClt(:,2) = CoClt(:,2)-GBkg;


%------------------------------------------------------------------------

% NORMALIZING FLUORESCENCE VALUES RELATIVE TO THE MEDIAN OF CONTROLS

% Determiminmg median Green = MedGren from Green Control
MedGren = median(GrCt(:,2));
```

```
% Determiminmg median Red = MedRed from Red Control
MedRed = median(RdCt(:,1));

% Normalizing control Red and Green values relative to medians
GrCt(:,6)=GrCt(:,2)*100/MedGren;
GrCt(:,5)=GrCt(:,1)*100/MedRed;
RdCt(:,6)=RdCt(:,2)*100/MedGren;
RdCt(:,5)=RdCt(:,1)*100/MedRed;
CoClt(:,6)=CoClt(:,2)*100/MedGren;
CoClt(:,5)=CoClt(:,1)*100/MedRed;

clear MedRed MedGren GBkg RBkg

%---------------------------------------------------------------------

% CALCULATION OF EXCHANGE UNTS FOR CELLS IN CO-CULTURES

% DEFINING CRITICAL LIMITS REQUIRED TO CLASSIFY CELLS
% Maximum Red in Green Controls = MaxRdinGreen
MaxRdinGreen = max(GrCt(:,5));

% Maximum Green in Red Controls = MaxGrinRed
MaxGrinRed = max(RdCt(:,6));

% Minimum Green in Green Controls = MinGrinGreen
MinGrinGreen = min(GrCt(:,6));

% Minimum Red in Red Controls = MinRdinRed
MinRdinRed = min(RdCt(:,5));


% CALCULATION OF THE ANGLE SUBTENDED FROM THE ORIGIN
% Angle in Radians from Inverse Tan of Red / Green
for i=1:cc
   CoClt(i,7)=atan(CoClt(i,5)/CoClt(i,6));
end


% IDENTIFYING CELLS ON THE BISECTING LINE AND ASSIGNING 0 EXCHANGE UNITS
for i=1:cc
   if CoClt(i,7) == pi/4
      CoClt(i,8) = 0;
   end
end

% DEFINING THE NUMBER OF RADIANS PER EXCHANGE UNIT = EU
EU = (pi/2)/100;


% IDENTIFYING CELLS ABOVE THE BISECTING LINE AND ASSIGNING NEGATIVE
% EXCHANGE UNITS
for i=1:cc
   if CoClt(i,7) > pi/4
      CoClt(i,8) = -((CoClt(i,7)-(pi/4))/EU);
```

```
    end
end


% IDENTIFYING CELLS BELOW THE BISECTING LINE AND ASSIGNING POSITIVE
% EXCHANGE UNITS
for i=1:cc
    if CoClt(i,7) < pi/4
        CoClt(i,8) = ((pi/4)-CoClt(i,7))/EU;
    end
end

% CLASSIFICATION OF CELLS AS BELONGING TO GROUPS 2, 3 OR 4
for i=1:cc
    % Group 2
    if CoClt(i,5) >= MinRdinRed & CoClt(i,6) < MinGrinGreen
        CoClt(i,9) = 2;
    end
    % Group 3
    if CoClt(i,5) >= MinRdinRed & CoClt(i,6) >= MinGrinGreen
        CoClt(i,9) = 3;
    end
    % Group 4
    if CoClt(i,5) < MinRdinRed & CoClt(i,6) >= MinGrinGreen
        CoClt(i,9) = 4;
    end
end

% IDENTIFYING CELLS INDISTINGUISHABLE FROM CONTROLS AND ASSIGNING BOTH
% EXCHANGE NUMBER AND GROUP CODE
% Cells indistinguishable from Red Controls
for i = 1:cc
    if CoClt(i,6) <= MaxGrinRed & CoClt(i,5) >= MinRdinRed
        CoClt(i,8) = -50;
        CoClt(i,9) = 1;
    end
end

% Cells indistinguishable from Green Controls
for i = 1:cc
    if CoClt(i,5) <= MaxRdinGreen & CoClt(i,6) >= MinGrinGreen
        CoClt(i,8) = 50;
        CoClt(i,9) = 5;
    end
end

%--------------------------------------------------------------------

% REMOVAL OF LOW OUTLIER CELLS FROM ANALYSIS AND CREATION OF A
% MATRIX FOR CO-CULTURE GRAPHING = CoGrph

% Counting cells with '0' group to determine rows needed for CoGrph
count = 0;
for i=1:cc
```

```
    if CoClt(i,9) == 0
        count = count + 1;
    end
end
rows = cc - count;

% Creation of matrix CoGrph
CoGrph = zeros (rows,9);

% Loading data into CoGrph
count = 1;

for i=1:cc
    if CoClt(i,9) > 0
       CoGrph(count,:) = CoClt(i,:);
       count = count + 1;
    else
        continue
    end
end

% Replacing Fluorescence Values with 0 where Exchange Units = -/+ 50
for i = 1:rows
    if CoGrph(i,9) == 1
        CoGrph(i,6) = 0;
    end
    if CoGrph(i,9) == 5
        CoGrph(i,5) = 0;
    end
end

clear cc CoClt count EU i MaxGrinRed MaxRdinGreen MinGrinGreen
clear MinRdinRed

% Saving CoGrph Data Matrix
dlmwrite('CoCultureData.txt',CoGrph,'Delimiter',';');

% Saving Green and Red Control Data Matrices
dlmwrite('GreenControlData.txt',GrCt,'Delimiter',';');
dlmwrite('RedControlData.txt',RdCt,'Delimiter',';');



%-----------------------------------------------------------------------

% PLOTTING RESULTS ON THE CARTESIAN PLANE

% Setting the font size for all graphs
set(0,'DefaultAxesFontSize',16);

% LOADING RESULTS INTO LISTS FOR PLOTTING
% Control Green Cells
szG = size(GrCt);
gRow = szG(1,1);
```

```
xContGreen = zeros(gRow,1);
xContGreen(:,1) = GrCt(:,6);
yContGreen = zeros(gRow,1);

%  Control Red Cells
szR = size(RdCt);
rRow = szR(1,1);
xContRed = zeros(rRow,1);
yContRed = zeros(rRow,1);
yContRed(:,1) = RdCt(:,5);

% Combining Control Cells into a Single Set - xCont & yCont
xCont = zeros ((gRow+rRow),1);
yCont = zeros ((gRow+rRow),1);
for i = 1:gRow
   xCont(i,1) = xContGreen(i,1);
   yCont(i,1) = yContGreen(i,1);
end

for i = 1:rRow
   xCont(gRow+i,1) = xContRed(i,1);
   yCont(gRow+i,1) = yContRed(i,1);
end

% Co-cultured Cells in Group 1
% Creating matrices, Loading values, Removing Empty values
xGrp1a = zeros(rows,1);
yGrp1a = zeros(rows,1);
for i = 1:rows
   if CoGrph(i,9)==1
      xGrp1a(i,1) = CoGrph(i,6);
      yGrp1a(i,1) = CoGrph(i,5);
   end
end
% Counting the number of values
count = 0;
for i = 1:rows
   if yGrp1a(i,1) > 0
      count = count +1;
   end
end
r = count;
% Creating final matrices and loading results
xGrp1 = zeros(r,1);
yGrp1 = zeros(r,1);
count = 1;
for i = 1:rows
   if yGrp1a(i,1) > 0
      yGrp1(count,1) = yGrp1a(i,1);
      xGrp1(count,1) = 0;
      count = count+1;
   end
end
```

```
clear xGrp1a yGrp1a cnt count gRow i r rRows szG szR


% Co-cultured Cells in Group 2
% Creating matrices, Loading values, Removing Empty values
xGrp2a = zeros(rows,1);
yGrp2a = zeros(rows,1);
for i = 1:rows
   if CoGrph(i,9)==2
      xGrp2a(i,1) = CoGrph(i,6);
      yGrp2a(i,1) = CoGrph(i,5);
   end
end
% Counting the number of values
count = 0;
for i = 1:rows
   if yGrp2a(i,1) > 0
      count = count +1;
   end
end
r = count;
% Creating final matrices and loading results
xGrp2 = zeros(r,1);
yGrp2 = zeros(r,1);
count = 1;
for i = 1:rows
   if yGrp2a(i,1) > 0
      yGrp2(count,1) = yGrp2a(i,1);
      xGrp2(count,1) = xGrp2a(i,1);
      count = count+1;
   end
end

clear xGrp2a yGrp2a cnt count i r

% Co-cultured Cells in Group 3
% Creating matrices, Loading values, Removing Empty values
xGrp3a = zeros(rows,1);
yGrp3a = zeros(rows,1);
for i = 1:rows
   if CoGrph(i,9)==3
      xGrp3a(i,1) = CoGrph(i,6);
      yGrp3a(i,1) = CoGrph(i,5);
   end
end
% Counting the number of values
count = 0;
for i = 1:rows
   if yGrp3a(i,1) > 0
      count = count +1;
   end
end
r = count;
% Creating final matrices and loading results
```

```
xGrp3 = zeros(r,1);
yGrp3 = zeros(r,1);
count = 1;
for i = 1:rows
    if yGrp3a(i,1) > 0
        yGrp3(count,1) = yGrp3a(i,1);
        xGrp3(count,1) = xGrp3a(i,1);
        count = count+1;
    end
end

clear xGrp3a yGrp3a cnt count i r


% Co-cultured Cells in Group 4
% Creating matrices, Loading values, Removing Empty values
xGrp4a = zeros(rows,1);
yGrp4a = zeros(rows,1);
for i = 1:rows
    if CoGrph(i,9)==4
        xGrp4a(i,1) = CoGrph(i,6);
        yGrp4a(i,1) = CoGrph(i,5);
    end
end
% Counting the number of values
count = 0;
for i = 1:rows
    if xGrp4a(i,1) > 0
        count = count +1;
    end
end
r = count;
% Creating final matrices and loading results
xGrp4 = zeros(r,1);
yGrp4 = zeros(r,1);
count = 1;
for i = 1:rows
    if xGrp4a(i,1) > 0
        yGrp4(count,1) = yGrp4a(i,1);
        xGrp4(count,1) = xGrp4a(i,1);
        count = count+1;
    end
end

clear xGrp4a yGrp4a cnt count i r


% Co-cultured Cells in Group 5
% Creating matrices, Loading values, Removing Empty values
xGrp5a = zeros(rows,1);
yGrp5a = zeros(rows,1);
for i = 1:rows
    if CoGrph(i,9)==5
        xGrp5a(i,1) = CoGrph(i,6);
```

```
    end
end
% Counting the number of values
count = 0;
for i = 1:rows
    if xGrp5a(i,1) > 0
        count = count +1;
    end
end
r = count;
% Creating final matrices and loading results
xGrp5 = zeros(r,1);
yGrp5 = zeros(r,1);
count = 1;
for i = 1:rows
    if xGrp5a(i,1) > 0
        xGrp5(count,1) = xGrp5a(i,1);
        count = count+1;
    end
end

clear xGrp5a yGrp5a cnt count i r rRow

% MAKING THE LINEAR CARTESIAN PLOT
% Plotting Control Cells
x = xCont;
y = yCont;
subplot(2,3,1);
xlim([0 xlinhi])
ylim([0 ylinhi])
s=60;
scatter(x,y,s,'^','MarkerEdgeColor',[0 0 0]);
hold on;

% Defining marker size for remaining components
s=10;

% Plotting Group 1 Cells
x = xGrp1;
y = yGrp1;
subplot(2,3,1);
xlim([0 xlinhi])
ylim([0 ylinhi])
scatter(x,y,s,'MarkerFaceColor',[1 0 0], 'MarkerEdgeColor',[1 0 0]);
hold on;

% Plotting Group 2 Cells
x = xGrp2;
y = yGrp2;
subplot(2,3,1);
xlim([0 xlinhi])
ylim([0 ylinhi])
scatter(x,y,s,'MarkerFaceColor',[0.95 0.4 0.1],...
    'MarkerEdgeColor',[0.95 0.4 0.1]);
```

```
hold on;

% Plotting Group 3 Cells
x = xGrp3;
y = yGrp3;
subplot(2,3,1);
xlim([0 xlinhi])
ylim([0 ylinhi])
scatter(x,y,s,'MarkerFaceColor',[0.9292 0.694 0.125],...
    'MarkerEdgeColor',[0.9292 0.694 0.125]);
hold on;

% Plotting Group 4 Cells
x = xGrp4;
y = yGrp4;
subplot(2,3,1);
xlim([0 xlinhi])
ylim([0 ylinhi])
scatter(x,y,s,'MarkerFaceColor',[0.2 0.9 0.1],...
    'MarkerEdgeColor',[0.2 0.9 0.1]);
hold on;

% Plotting Group 5 Cells
x = xGrp5;
y = yGrp5;
subplot(2,3,1);
xlim([0 xlinhi])
ylim([0 ylinhi])
scatter(x,y,s,'MarkerFaceColor',[0 0.5 0],...
    'MarkerEdgeColor',[0 0.5 0]);

% Labelling Axes
title('Cartesian Plot')
xlabel('Green Fluorescence')
ylabel('Red Fluorecence')

% MAKING THE LOG LOG CARTESIAN GRAPH
% Plotting Red Controls
xlog = xCont;
xlog(:,1) = lgx;
x = xlog;
y = yCont;
subplot(2,3,2);
xlim([xlglow xlghi])
ylim([ylglow ylghi])
s=60;
scatter(x,y,s,'^','MarkerEdgeColor',[0 0 0]);
set(gca,'xscale','log')
set(gca,'yscale','log')
hold on;

% Plotting Green Controls
x = xCont;
ylog = yCont;
```

```
ylog(:,1) = lgy;
y = ylog;
subplot(2,3,2);
xlim([xlglow xlghi])
ylim([ylglow ylghi])
s=60;
scatter(x,y,s,'^','MarkerEdgeColor',[0 0 0]);
set(gca,'xscale','log')
set(gca,'yscale','log')
hold on;

% Defining marker size for remaining components
s=10;

% Plotting Group 1 Cells
redlog = xGrp1;
redlog(:,1) = lgx;
x = redlog;
y = yGrp1;
subplot(2,3,2);
xlim([xlglow xlghi])
ylim([ylglow ylghi])
scatter(x,y,s,'MarkerFaceColor',[1 0 0], 'MarkerEdgeColor',[1 0 0]);
set(gca,'xscale','log')
set(gca,'yscale','log')
hold on;

% Plotting Group 2 Cells
x = xGrp2;
y = yGrp2;
subplot(2,3,2);
xlim([xlglow xlghi])
ylim([ylglow ylghi])
scatter(x,y,s,'MarkerFaceColor',[0.95 0.4 0.1],...
    'MarkerEdgeColor',[0.95 0.4 0.1]);
set(gca,'xscale','log')
set(gca,'yscale','log')
hold on;

% Plotting Group 3 Cells
x = xGrp3;
y = yGrp3;
subplot(2,3,2);
xlim([xlglow xlghi])
ylim([ylglow ylghi])
scatter(x,y,s,'MarkerFaceColor',[0.9292 0.694 0.125],...
    'MarkerEdgeColor',[0.9292 0.694 0.125]);
set(gca,'xscale','log')
set(gca,'yscale','log')
hold on;

% Plotting Group 4 Cells
x = xGrp4;
y = yGrp4;
```

```
subplot(2,3,2);
xlim([xlglow xlghi])
ylim([ylglow ylghi])
scatter(x,y,s,'MarkerFaceColor',[0.2 0.9 0.1],...
    'MarkerEdgeColor',[0.2 0.9 0.1]);
set(gca,'xscale','log')
set(gca,'yscale','log')
hold on;

% Plotting Group 5 Cells

greenlog = yGrp5;
greenlog(:,1) = lgy;
x = xGrp5;
y = greenlog;
subplot(2,3,2);
xlim([xlglow xlghi])
ylim([ylglow ylghi])
scatter(x,y,s,'MarkerFaceColor',[0 0.5 0],...
    'MarkerEdgeColor',[0 0.5 0]);
set(gca,'xscale','log')
set(gca,'yscale','log')
hold on;

% Labelling Axes
title('Cartesian Plot')
xlabel('Green Fluorescence')
ylabel('Red Fluorecence')

% Constructing the Legend
lbl = {'Control Green Cells',...
    'Control Red Cells',...
    'Co-Cultured Cells Indistinguishable from Red Controls',...
    'Co-Cultured Red Cells with Some Green',...
    'Co-Cultured Cells of Uncertain Origin',...
    'Co-Cultured Green Cells with Some Red',...
    'Co-Cultured Cells Indistinguishable from Green Controls'};
legend('o',lbl,'Location','EastOutside');
hold on;

clear s x xCoClt xCont xContGreen xContRed xGrp1 xGrp2 xGrp3
clear xGrp4 xGrp5 y yCont yContGreen yContRed yGrp1 yGrp1 yGrp2
clear yGrp3 yGrp4 yGrp5 lgx lgy redlog xlghi xlglow xlinhi
clear xlog ylghi ylglow ylinhi ylog greenlog


%----------------------------------------------------------------------
% SCATTERPLOT FOR CIRCULARITY AGAINST EXCHANGE UNITS

% LOADING RESULTS INTO LISTS FOR PLOTTING
% Control Green Cells
szG = size(GrCt);
gRow = szG(1,1);
xContGreen = zeros(gRow,1);
```

```
xContGreen(:,1) = 50;
yContGreen = zeros(gRow,1);
yContGreen = GrCt(:,3);

%  Control Red Cells
szR = size(RdCt);
rRow = szR(1,1);
xContRed = zeros(rRow,1);
xContRed(:,1) = -50;
yContRed = zeros(rRow,1);
yContRed(:,1) = RdCt(:,3);

% Combining Control Cells into a Single Set - xCont & yCont
xCont = zeros ((gRow+rRow),1);
yCont = zeros ((gRow+rRow),1);
for i = 1:gRow
   xCont(i,1) = xContGreen(i,1);
   yCont(i,1) = yContGreen(i,1);
end

for i = 1:rRow
   xCont(gRow+i,1) = xContRed(i,1);
   yCont(gRow+i,1) = yContRed(i,1);
end


% Matrices for Control Circiularity;
CircRedControl = RdCt(:,3);
CircGreenControl = GrCt(:,3);


% Co-cultured Cells in Group 1
% Creating matrices, Loading values, Removing Empty values
xGrp1a = zeros(rows,1);
yGrp1a = zeros(rows,1);
for i = 1:rows
   if CoGrph(i,9)==1
      xGrp1a(i,1) = -50;
      yGrp1a(i,1) = CoGrph(i,3);
   end
end
% Counting the number of values
count = 0;
for i = 1:rows
   if yGrp1a(i,1) > 0
      count = count +1;
   end
end
r = count;
% Creating final matrices and loading results
xGrp1 = zeros(r,1);
yGrp1 = zeros(r,1);
count = 1;
for i = 1:rows
```

```
    if yGrp1a(i,1) > 0
        yGrp1(count,1) = yGrp1a(i,1);
        xGrp1(count,1) = -50;
        count = count+1;
    end
end

clear xGrp1a yGrp1a cnt count gRow i r rRows szG szR


% Co-cultured Cells in Group 2
% Creating matrices, Loading values, Removing Empty values
xGrp2a = zeros(rows,1);
yGrp2a = zeros(rows,1);
for i = 1:rows
    if CoGrph(i,9)==2
        xGrp2a(i,1) = CoGrph(i,8);
        yGrp2a(i,1) = CoGrph(i,3);
    end
end
% Counting the number of values
count = 0;
for i = 1:rows
    if yGrp2a(i,1) > 0
        count = count +1;
    end
end
r = count;
% Creating final matrices and loading results
xGrp2 = zeros(r,1);
yGrp2 = zeros(r,1);
count = 1;
for i = 1:rows
    if yGrp2a(i,1) > 0
        yGrp2(count,1) = yGrp2a(i,1);
        xGrp2(count,1) = xGrp2a(i,1);
        count = count+1;
    end
end

clear xGrp2a yGrp2a cnt count i r


% Co-cultured Cells in Group 3
% Creating matrices, Loading values, Removing Empty values
xGrp3a = zeros(rows,1);
yGrp3a = zeros(rows,1);
for i = 1:rows
    if CoGrph(i,9)==3
        xGrp3a(i,1) = CoGrph(i,8);
        yGrp3a(i,1) = CoGrph(i,3);
    end
end
% Counting the number of values
```

```
count = 0;
for i = 1:rows
    if yGrp3a(i,1) > 0
        count = count +1;
    end
end
r = count;
% Creating final matrices and loading results
xGrp3 = zeros(r,1);
yGrp3 = zeros(r,1);
count = 1;
for i = 1:rows
    if yGrp3a(i,1) > 0
        yGrp3(count,1) = yGrp3a(i,1);
        xGrp3(count,1) = xGrp3a(i,1);
        count = count+1;
    end
end

clear xGrp3a yGrp3a cnt count i r


% Co-cultured Cells in Group 4
% Creating matrices, Loading values, Removing Empty values
xGrp4a = zeros(rows,1);
yGrp4a = zeros(rows,1);
for i = 1:rows
    if CoGrph(i,9)==4
        xGrp4a(i,1) = CoGrph(i,8);
        yGrp4a(i,1) = CoGrph(i,3);
    end
end
% Counting the number of values
count = 0;
for i = 1:rows
    if xGrp4a(i,1) > 0
        count = count +1;
    end
end
r = count;
% Creating final matrices and loading results
xGrp4 = zeros(r,1);
yGrp4 = zeros(r,1);
count = 1;
for i = 1:rows
    if xGrp4a(i,1) > 0
        yGrp4(count,1) = yGrp4a(i,1);
        xGrp4(count,1) = xGrp4a(i,1);
        count = count+1;
    end
end

clear xGrp4a yGrp4a cnt count i r
```

```
% Co-cultured Cells in Group 5
% Creating matrices, Loading values, Removing Empty values
xGrp5a = zeros(rows,1);
yGrp5a = zeros(rows,1);
for i = 1:rows
   if CoGrph(i,9)==5
      xGrp5a(i,1) =50;
      yGrp5a(i,1) = CoGrph(i,3);
   end
end
% Counting the number of values
count = 0;
for i = 1:rows
   if xGrp5a(i,1) > 0
      count = count +1;
   end
end
r = count;
% Creating final matrices and loading results
xGrp5 = zeros(r,1);
yGrp5 = zeros(r,1);
count = 1;
for i = 1:rows
   if xGrp5a(i,1) > 0
      yGrp5(count,1) = yGrp5a(i,1);
      xGrp5(count,1) = xGrp5a(i,1);
      count = count+1;
   end
end

clear xGrp5a yGrp5a cnt count i r rRow

% Creating a Blank Matrix to Accept Group Circularity Data in the Order:
  % Column 1: Control Red Cells
  % Column 2: Group 1
  % Column 3: Group 2
  % Column 4: Group 3
  % Column 5: Group 4
  % Column 6: Group 5
  % Column 7: Control Green Cells
% And Loading and Saving as CircGroupData.txt

CircGroupData = zeros(cMAX,7);

sz = size (CircRedControl);
l = sz(1,1);
CircGroupData(1:l,1) = CircRedControl(1:l,1);

sz = size (yGrp1);
l = sz(1,1);
CircGroupData(1:l,2) = yGrp1(1:l,1);

sz = size (yGrp2);
l = sz(1,1);
```

```
CircGroupData(1:l,3) = yGrp2(1:l,1);

sz = size (yGrp3);
l = sz(1,1);
CircGroupData(1:l,4) = yGrp3(1:l,1);

sz = size (yGrp4);
l = sz(1,1);
CircGroupData(1:l,5) = yGrp4(1:l,1);

sz = size (yGrp5);
l = sz(1,1);
CircGroupData(1:l,6) = yGrp5(1:l,1);

sz = size (CircGreenControl);
l = sz(1,1);
CircGroupData(1:l,7) = CircGreenControl(1:l,1);

% Saving Circularity Group Data Matrix
dlmwrite('CircGroupData.txt',CircGroupData,'Delimiter',';');

clear l sz CircGroupData

% CREATING AND LOADING A MATRIX FOR GROUP CIRCULARITY MEDIANS
MedianGroupCircularity = zeros (1,7);
MedianGroupCircularity (1,1) =  median(RdCt(:,3));
MedianGroupCircularity (1,2) = median(yGrp1);
MedianGroupCircularity (1,3) = median(yGrp2);
MedianGroupCircularity (1,4) = median(yGrp3);
MedianGroupCircularity (1,5) = median(yGrp4);
MedianGroupCircularity (1,6) = median(yGrp5);
MedianGroupCircularity (1,7) = median(GrCt(:,3));

% Saving Median Circularity Group Data Matrix
dlmwrite('MedianGroupCircularity.txt',MedianGroupCircularity,...
    'Delimiter',';');


% DETERMINING MEDIAN CIRCULARITY ACCORDING TO EU FOR PLOTTING
% Creating Matrix to Record Median Circularity
MedianEUCircularity = zeros(1,9);

% Median Circularity of Control Cells (yMedGreenCont, yMedRedCont)
yMedGreenCont = median(GrCt(:,3));
xMedGreenCont = 50;
yMedRedCont = median(RdCt(:,3));
xMedRedCont = -50;

% Loading Median Control Circularity into Matrix
MedianEUCircularity (1,1) = yMedRedCont;
MedianEUCircularity (1,9) = yMedGreenCont;


% Finding Median  Circularity for Exchange Units -50 (Group 1)
```

```
yMedianCircNeg50 = median(yGrp1);

% Loading Median Circularity into Matrix
MedianEUCircularity (1,2) = yMedianCircNeg50;


% Finding Median Circularity for Exchange Units -50 to -30
yNeg50toNeg30a = zeros(rows,1);
for i = 1:rows
   if CoGrph(i,8)>-50 && CoGrph(i,8)<=-30
      yNeg50toNeg30a(i,1) = CoGrph(i,3);
   end
end
% Counting the number of values
count = 0;
for i = 1:rows
   if yNeg50toNeg30a(i,1) > 0
      count = count +1;
   end
end
r = count;
% Creating final matrices and loading results
yNeg50toNeg30b = zeros(r,1);
count = 1;
for i = 1:rows
   if yNeg50toNeg30a(i,1) > 0
      yNeg50toNeg30b(count,1) = yNeg50toNeg30a(i,1);
      count = count+1;
   end
end
% Finding Median and loading results
yNeg50toNeg30 = median(yNeg50toNeg30b(:,1));
xNeg50toNeg30 = -40;

% Loading Median Circularity into Matrix
MedianEUCircularity (1,3) = yNeg50toNeg30;

% Finding Median Circularity for Exchange Units -30 to -10
yNeg30toNeg10a = zeros(rows,1);
for i = 1:rows
   if CoGrph(i,8)>-30 && CoGrph(i,8)<=-10
      yNeg30toNeg10a(i,1) = CoGrph(i,3);
   end
end
% Counting the number of values
count = 0;
for i = 1:rows
   if yNeg30toNeg10a(i,1) > 0
      count = count +1;
   end
end
r = count;
% Creating final matrices and loading results
yNeg30toNeg10b = zeros(r,1);
```

```
count = 1;
for i = 1:rows
   if yNeg30toNeg10a(i,1) > 0
      yNeg30toNeg10b(count,1) = yNeg30toNeg10a(i,1);
      count = count+1;
   end
end
% Finding Median and loading results
yNeg30toNeg10 = median(yNeg30toNeg10b(:,1));
xNeg30toNeg10 = -20;

% Loading Median Circularity into Matrix
MedianEUCircularity (1,4) = yNeg30toNeg10;

% Finding Median Circularity for Exchange Units -10 to 10
yNeg10toPos10a = zeros(rows,1);
for i = 1:rows
   if CoGrph(i,8)>-10 && CoGrph(i,8)<=10
      yNeg10toPos10a(i,1) = CoGrph(i,3);
   end
end
% Counting the number of values
count = 0;
for i = 1:rows
   if yNeg10toPos10a(i,1) > 0
      count = count +1;
   end
end
r = count;
% Creating final matrices and loading results
yNeg10toPos10b = zeros(r,1);
count = 1;
for i = 1:rows
   if yNeg10toPos10a(i,1) > 0
      yNeg10toPos10b(count,1) = yNeg10toPos10a(i,1);
      count = count+1;
   end
end
% Finding Median and loading results
yNeg10toPos10 = median(yNeg10toPos10b(:,1));
xNeg10toPos10 = 0;

% Loading Median Circularity into Matrix
MedianEUCircularity (1,5) = yNeg10toPos10;

% Finding Median Circularity for Exchange Units 10 to 30
yPos10toPos30a = zeros(rows,1);
for i = 1:rows
   if CoGrph(i,8)>10 && CoGrph(i,8)<=30
      yPos10toPos30a(i,1) = CoGrph(i,3);
   end
end
% Counting the number of values
count = 0;
```

```
for i = 1:rows
    if yPos10toPos30a(i,1) > 0
        count = count +1;
    end
end
r = count;
% Creating final matrices and loading results
yPos10toPos30b = zeros(r,1);
count = 1;
for i = 1:rows
    if yPos10toPos30a(i,1) > 0
        yPos10toPos30b(count,1) = yPos10toPos30a(i,1);
        count = count+1;
    end
end
% Finding Median and loading results
yPos10toPos30 = median(yPos10toPos30b(:,1));
xPos10toPos30 = 20;

% Loading Median Circularity into Matrix
MedianEUCircularity (1,6) = yPos10toPos30;

% Finding Median Circularity for Exchange Units 30 to 50
yPos30toPos50a = zeros(rows,1);
for i = 1:rows
    if CoGrph(i,8)>30 && CoGrph(i,8)<50
        yPos30toPos50a(i,1) = CoGrph(i,3);
    end
end
% Counting the number of values
count = 0;
for i = 1:rows
    if yPos30toPos50a(i,1) > 0
        count = count +1;
    end
end
r = count;
% Creating final matrices and loading results
yPos30toPos50b = zeros(r,1);
count = 1;
for i = 1:rows
    if yPos30toPos50a(i,1) > 0
        yPos30toPos50b(count,1) = yPos30toPos50a(i,1);
        count = count+1;
    end
end


% Creating a Blank Matrix to Accept EU Circularity Data in the Order:
   % Column 1: Control Red Cells
   % Column 2: Group 1
   % Column 3: Neg50toNeg30b
   % Column 4: Neg30toNeg10b
   % Column 5: Neg10toPos10b
```

```
  % Column 6: Pos10toPos30b
  % Column 7: Pos30toPos50b
  % Column 8: Group 5
  % Column 9: Control Green Cells
% And Loading and Saving as CircEUData.txt

CircEUData = zeros(cMAX,9);

sz = size (CircRedControl);
l = sz(1,1);
CircEUData(1:l,1) = CircRedControl(1:l,1);

sz = size (yGrp1);
l = sz(1,1);
CircEUData(1:l,2) = yGrp1(1:l,1);

sz = size (yNeg50toNeg30b);
l = sz(1,1);
CircEUData(1:l,3) = yNeg50toNeg30b(1:l,1);

sz = size (yNeg30toNeg10b);
l = sz(1,1);
CircEUData(1:l,4) = yNeg30toNeg10b(1:l,1);

sz = size (yNeg10toPos10b);
l = sz(1,1);
CircEUData(1:l,5) = yNeg10toPos10b(1:l,1);

sz = size (yPos10toPos30b);
l = sz(1,1);
CircEUData(1:l,6) = yPos10toPos30b(1:l,1);

sz = size (yPos30toPos50b);
l = sz(1,1);
CircEUData(1:l,7) = yPos30toPos50b(1:l,1);

sz = size (yGrp5);
l = sz(1,1);
CircEUData(1:l,8) = yGrp5(1:l,1);

sz = size (CircGreenControl);
l = sz(1,1);
CircEUData(1:l,9) = CircGreenControl(1:l,1);

% Saving Circularity EU Data Matrix
dlmwrite('CircEUData.txt',CircEUData,'Delimiter',';');

clear l sz CircEUData

% Finding Median and loading results
yPos30toPos50 = median(yPos30toPos50b(:,1));
xPos30toPos50 = 40;

% Loading Median Circularity into Matrix
```

```
MedianEUCircularity (1,7) = yPos30toPos50;

% Finding Median  Circularity for Exchange Units 50 (Group 5)
yMedianCircPos50 = median(yGrp5);

% Loading Median Circularity into Matrix
MedianEUCircularity (1,8) = yMedianCircPos50;


% Saving Median Matrix
dlmwrite('MedianEUCircularity.txt',MedianEUCircularity,'Delimiter',';')

clear count i r yNeg50toNeg30a yNeg10toPos10b yNeg30toNeg10a
clear yNeg10toPos10a yPos10toPos30a yPos30toPos50a MedianEUCircularity


% MAKING THE PLOT
% Plotting Control Cells
x = xCont;
y = yCont;
subplot(2,3,4);
ylim([0 1])
s=60;
scatter(x,y,s,'^','MarkerEdgeColor',[0 0 0]);
hold on;

% Defining marker size
s=10;

% Plotting Group 1 Cells
x = xGrp1;
y = yGrp1;
subplot(2,3,4);
ylim([0 1])
scatter(x,y,s,'MarkerFaceColor',[1 0 0], 'MarkerEdgeColor',[1 0 0]);
hold on;

% Plotting Group 2 Cells
x = xGrp2;
y = yGrp2;
subplot(2,3,4);
ylim([0 1])
scatter(x,y,s,'MarkerFaceColor',[0.95 0.4 0.1],...
    'MarkerEdgeColor',[0.95 0.4 0.1]);
hold on;

% Plotting Group 3 Cells
x = xGrp3;
y = yGrp3;
subplot(2,3,4);
ylim([0 1])
scatter(x,y,s,'MarkerFaceColor',[0.9292 0.694 0.125],...
    'MarkerEdgeColor',[0.9292 0.694 0.125]);
hold on;
```

```
% Plotting Group 4 Cells
x = xGrp4;
y = yGrp4;
subplot(2,3,4);
ylim([0 1])
scatter(x,y,s,'MarkerFaceColor',[0.2 0.9 0.1],...
    'MarkerEdgeColor',[0.2 0.9 0.1]);
hold on;

% Plotting Group 5 Cells
x = xGrp5;
y = yGrp5;
subplot(2,3,4);
ylim([0 1])
scatter(x,y,s,'MarkerFaceColor',[0 0.5 0],...
    'MarkerEdgeColor',[0 0.5 0]);

% Labelling the scattergram
title('Circularity')
xlabel('Exchange Units')
ylabel('Circularity')
hold on;

clear s x xCoClt xCont xContGreen xContRed xGrp1 xGrp2 xGrp3
clear xGrp4 xGrp5 y yCont yContGreen yContRed yGrp1 yGrp1 yGrp2
clear yGrp3 yGrp4 yGrp5 cmap ii lbl p

% Defining Triangle Size for Control Median Plots
s = 250;

% Plotting Median Value Circularity Control Green
x = xMedGreenCont;
y = yMedGreenCont;
subplot(2,3,4);
scatter(x,y,s,'^','MarkerFaceColor',[0 0.5 0],...
    'MarkerEdgeColor',[0 0 0]);
hold on;

% Plotting Median Value Circularity Control Red
x = xMedRedCont;
y = yMedRedCont;
subplot(2,3,4);
scatter(x,y,s,'^','MarkerFaceColor',[1 0 0],...
    'MarkerEdgeColor',[0 0 0]);
hold on;

% Defining Square Size for Co-Culture Median Plots
s = 150;

% Plotting Median Value Circularity -50
x = -50;
y = yMedianCircNeg50;
subplot(2,3,4);
```

```
scatter(x,y,s,'s','MarkerFaceColor',[0 0 0],...
    'MarkerEdgeColor',[0 0 0]);
hold on;

% Plotting Median Value Circularity -50 to -30
x = xNeg50toNeg30;
y = yNeg50toNeg30;
subplot(2,3,4);
scatter(x,y,s,'s','MarkerFaceColor',[0 0 0],...
    'MarkerEdgeColor',[0 0 0]);
hold on;

% Plotting Median Value Circularity -30 to -10
x = xNeg30toNeg10;
y = yNeg30toNeg10;
subplot(2,3,4);
scatter(x,y,s,'s','MarkerFaceColor',[0 0 0],...
    'MarkerEdgeColor',[0 0 0]);
hold on;

% Plotting Median Value Circularity -10 to 10
x = xNeg10toPos10;
y = yNeg10toPos10;
subplot(2,3,4);
scatter(x,y,s,'s','MarkerFaceColor',[0 0 0],...
    'MarkerEdgeColor',[0 0 0]);
hold on;

% Plotting Median Value Circularity 10 to 30
x = xPos10toPos30;
y = yPos10toPos30;
subplot(2,3,4);
scatter(x,y,s,'s','MarkerFaceColor',[0 0 0],...
    'MarkerEdgeColor',[0 0 0]);
hold on;

% Plotting Median Value Circularity 30 to 50
x = xPos30toPos50;
y = yPos30toPos50;
subplot(2,3,4);
scatter(x,y,s,'s','MarkerFaceColor',[0 0 0],...
    'MarkerEdgeColor',[0 0 0]);
hold on;

% Plotting Median Value Circularity 50
x = 50;
y = yMedianCircPos50;
subplot(2,3,4);
scatter(x,y,s,'s','MarkerFaceColor',[0 0 0],...
    'MarkerEdgeColor',[0 0 0]);
hold on;

% Labelling the scattergram
title('Circularity')
```

```
xlabel('Exchange Units')
ylabel('Circularity')
hold on;

clear s x xCoClt xCont xContGreen xContRed xGrp1 xGrp2 xGrp3
clear xGrp4 xGrp5 y yCont yContGreen yContRed yGrp1 yGrp1 yGrp2
clear yGrp3 yGrp4 yGrp5 xMedGreenCont xRedCont xNeg10toPos10
clear xNeg30toNeg10 xNeg50toNeg30 xPos10toPos30 xPos30toPos50
clear yMedGreenCont yNeg10toPos10 yNeg30toNeg10 yNeg30toNeg10b
clear yNeg50toNeg30 yNeg50toNeg30b yPos10toPos30 yPos10toPos30b
clear yPos30toPos50 yPos30toPos50b xMedRedCont yMedRedCont
clear yMedianCircNeg50 yMedianCircPos50 xNeg50 xPos50
clear CircGreenControl CircRedControl MedianGroupCircularity


%-------------------------------------------------------------------
% SCATTERPLOT FOR AREA AGAINST EXCHANGE NUMBER

% LOADING RESULTS INTO LISTS FOR PLOTTING
% Control Green Cells
szG = size(GrCt);
gRow = szG(1,1);
xContGreen = zeros(gRow,1);
xContGreen(:,1) = 50;
yContGreen = zeros(gRow,1);
yContGreen = GrCt(:,4);

%  Control Red Cells
szR = size(RdCt);
rRow = szR(1,1);
xContRed = zeros(rRow,1);
xContRed(:,1) = -50;
yContRed = zeros(rRow,1);
yContRed(:,1) = RdCt(:,4);

% Combining Control Cells into a Single Set - xCont & yCont
xCont = zeros ((gRow+rRow),1);
yCont = zeros ((gRow+rRow),1);
for i = 1:gRow
   xCont(i,1) = xContGreen(i,1);
   yCont(i,1) = yContGreen(i,1);
end

for i = 1:rRow
   xCont(gRow+i,1) = xContRed(i,1);
   yCont(gRow+i,1) = yContRed(i,1);
end

% Co-cultured Cells in Group 1
% Creating matrices, Loading values, Removing Empty values
xGrp1a = zeros(rows,1);
yGrp1a = zeros(rows,1);
for i = 1:rows
   if CoGrph(i,9)==1
```

```
        xGrp1a(i,1) = -50;
        yGrp1a(i,1) = CoGrph(i,4);
    end
end
% Counting the number of values
count = 0;
for i = 1:rows
    if yGrp1a(i,1) > 0
        count = count +1;
    end
end
r = count;
% Creating final matrices and loading results
xGrp1 = zeros(r,1);
yGrp1 = zeros(r,1);
count = 1;
for i = 1:rows
    if yGrp1a(i,1) > 0
        yGrp1(count,1) = yGrp1a(i,1);
        xGrp1(count,1) = -50;
        count = count+1;
    end
end

clear xGrp1a yGrp1a cnt count gRow i r rRows szG szR


% Co-cultured Cells in Group 2
% Creating matrices, Loading values, Removing Empty values
xGrp2a = zeros(rows,1);
yGrp2a = zeros(rows,1);
for i = 1:rows
    if CoGrph(i,9)==2
        xGrp2a(i,1) = CoGrph(i,8);
        yGrp2a(i,1) = CoGrph(i,4);
    end
end
% Counting the number of values
count = 0;
for i = 1:rows
    if yGrp2a(i,1) > 0
        count = count +1;
    end
end
r = count;
% Creating final matrices and loading results
xGrp2 = zeros(r,1);
yGrp2 = zeros(r,1);
count = 1;
for i = 1:rows
    if yGrp2a(i,1) > 0
        yGrp2(count,1) = yGrp2a(i,1);
        xGrp2(count,1) = xGrp2a(i,1);
        count = count+1;
```

```
    end
end

clear xGrp2a yGrp2a cnt count i r


% Co-cultured Cells in Group 3
% Creating matrices, Loading values, Removing Empty values
xGrp3a = zeros(rows,1);
yGrp3a = zeros(rows,1);
for i = 1:rows
    if CoGrph(i,9)==3
        xGrp3a(i,1) = CoGrph(i,8);
        yGrp3a(i,1) = CoGrph(i,4);
    end
end
% Counting the number of values
count = 0;
for i = 1:rows
    if yGrp3a(i,1) > 0
        count = count +1;
    end
end
r = count;
% Creating final matrices and loading results
xGrp3 = zeros(r,1);
yGrp3 = zeros(r,1);
count = 1;
for i = 1:rows
    if yGrp3a(i,1) > 0
        yGrp3(count,1) = yGrp3a(i,1);
        xGrp3(count,1) = xGrp3a(i,1);
        count = count+1;
    end
end

clear xGrp3a yGrp3a cnt count i r


% Co-cultured Cells in Group 4
% Creating matrices, Loading values, Removing Empty values
xGrp4a = zeros(rows,1);
yGrp4a = zeros(rows,1);
for i = 1:rows
    if CoGrph(i,9)==4
        xGrp4a(i,1) = CoGrph(i,8);
        yGrp4a(i,1) = CoGrph(i,4);
    end
end
% Counting the number of values
count = 0;
for i = 1:rows
    if xGrp4a(i,1) > 0
        count = count +1;
```

```
    end
end
r = count;
% Creating final matrices and loading results
xGrp4 = zeros(r,1);
yGrp4 = zeros(r,1);
count = 1;
for i = 1:rows
    if xGrp4a(i,1) > 0
        yGrp4(count,1) = yGrp4a(i,1);
        xGrp4(count,1) = xGrp4a(i,1);
        count = count+1;
    end
end

clear xGrp4a yGrp4a cnt count i r


% Co-cultured Cells in Group 5
% Creating matrices, Loading values, Removing Empty values
xGrp5a = zeros(rows,1);
yGrp5a = zeros(rows,1);
for i = 1:rows
    if CoGrph(i,9)==5
        xGrp5a(i,1) =50;
        yGrp5a(i,1) = CoGrph(i,4);
    end
end
% Counting the number of values
count = 0;
for i = 1:rows
    if xGrp5a(i,1) > 0
        count = count +1;
    end
end
r = count;
% Creating final matrices and loading results
xGrp5 = zeros(r,1);
yGrp5 = zeros(r,1);
count = 1;
for i = 1:rows
    if xGrp5a(i,1) > 0
        yGrp5(count,1) = yGrp5a(i,1);
        xGrp5(count,1) = xGrp5a(i,1);
        count = count+1;
    end
end

clear xGrp5a yGrp5a cnt count i r rRow


% Creating a Blank Matrix to Accept Group Area Data in the Order:
   % Column 1: Control Red Cells
   % Column 2: Group 1
```

```
  % Column 3: Group 2
  % Column 4: Group 3
  % Column 5: Group 4
  % Column 6: Group 5
  % Column 7: Control Green Cells
% And Loading and Saving as AreaGroupData.txt

AreaGroupData = zeros(cMAX,7);

sz = size (yContRed);
l = sz(1,1);
AreaGroupData(1:l,1) = yContRed(1:l,1);

sz = size (yGrp1);
l = sz(1,1);
AreaGroupData(1:l,2) = yGrp1(1:l,1);

sz = size (yGrp2);
l = sz(1,1);
AreaGroupData(1:l,3) = yGrp2(1:l,1);

sz = size (yGrp3);
l = sz(1,1);
AreaGroupData(1:l,4) = yGrp3(1:l,1);

sz = size (yGrp4);
l = sz(1,1);
AreaGroupData(1:l,5) = yGrp4(1:l,1);

sz = size (yGrp5);
l = sz(1,1);
AreaGroupData(1:l,6) = yGrp5(1:l,1);

sz = size (yContGreen);
l = sz(1,1);
AreaGroupData(1:l,7) = yContGreen(1:l,1);

% Saving Circularity Group Data Matrix
dlmwrite('AreaGroupData.txt',AreaGroupData,'Delimiter',';');

clear l sz AreaGroupData


% CREATING AND LOADING A MATRIX FOR GROUP AREA MEDIANS
MedianGroupArea = zeros (1,7);
MedianGroupArea (1,1) = median(RdCt(:,4));
MedianGroupArea (1,2) = median(yGrp1);
MedianGroupArea (1,3) = median(yGrp2);
MedianGroupArea (1,4) = median(yGrp3);
MedianGroupArea (1,5) = median(yGrp4);
MedianGroupArea (1,6) = median(yGrp5);
MedianGroupArea (1,7) = median(GrCt(:,4));

% Saving Median Area Group Data Matrix
```

```
dlmwrite('MedianGroupArea.txt',MedianGroupArea,...
  'Delimiter',';');


% DETERMINING MEDIAN AREA VALUES ACCORDING TO EU FOR PLOTTING
% Creating Matrix to Record Median Area
MedianEUArea = zeros(1,9);

% Median Area of Control Cells (yMedGreenCont, yMedRedCont)
yMedGreenCont = median(GrCt(:,4));
xMedGreenCont = 50;
yMedRedCont = median(RdCt(:,4));
xMedRedCont = -50;

% Loading Median Control Area into Matrix
MedianEUArea (1,1) = yMedRedCont;
MedianEUArea (1,9) = yMedGreenCont;

% Finding Median Area for Exchange Units -50 (Group 1)
yMedianEUAreaNeg50 = median(yGrp1);

% Loading Median Area into Matrix
MedianEUArea (1,2) = yMedianEUAreaNeg50;

% Finding Median Area for Exchange Units -50 to -30
yNeg50toNeg30a = zeros(rows,1);
for i = 1:rows
  if CoGrph(i,8)>-50 && CoGrph(i,8)<=-30
    yNeg50toNeg30a(i,1) = CoGrph(i,4);
  end
end
% Counting the number of values
count = 0;
for i = 1:rows
  if yNeg50toNeg30a(i,1) > 0
    count = count +1;
  end
end
r = count;
% Creating final matrices and loading results
yNeg50toNeg30b = zeros(r,1);
count = 1;
for i = 1:rows
  if yNeg50toNeg30a(i,1) > 0
    yNeg50toNeg30b(count,1) = yNeg50toNeg30a(i,1);
    count = count+1;
  end
end
% Finding Median and loading results
yNeg50toNeg30 = median(yNeg50toNeg30b(:,1));
xNeg50toNeg30 = -40;

% Loading Median Area into Matrix
MedianEUArea (1,3) = yNeg50toNeg30;
```

```
% Finding Median Area for Exchange Units -30 to -10
yNeg30toNeg10a = zeros(rows,1);
for i = 1:rows
   if CoGrph(i,8)>-30 && CoGrph(i,8)<=-10
      yNeg30toNeg10a(i,1) = CoGrph(i,4);
   end
end
% Counting the number of values
count = 0;
for i = 1:rows
   if yNeg30toNeg10a(i,1) > 0
      count = count +1;
   end
end
r = count;
% Creating final matrices and loading results
yNeg30toNeg10b = zeros(r,1);
count = 1;
for i = 1:rows
   if yNeg30toNeg10a(i,1) > 0
      yNeg30toNeg10b(count,1) = yNeg30toNeg10a(i,1);
      count = count+1;
   end
end
% Finding Median and loading results
yNeg30toNeg10 = median(yNeg30toNeg10b(:,1));
xNeg30toNeg10 = -20;

% Loading Median Area into Matrix
MedianEUArea (1,4) = yNeg30toNeg10;

% Finding Median Area for Exchange Units -10 to 10
yNeg10toPos10a = zeros(rows,1);
for i = 1:rows
   if CoGrph(i,8)>-10 && CoGrph(i,8)<=10
      yNeg10toPos10a(i,1) = CoGrph(i,4);
   end
end
% Counting the number of values
count = 0;
for i = 1:rows
   if yNeg10toPos10a(i,1) > 0
      count = count +1;
   end
end
r = count;
% Creating final matrices and loading results
yNeg10toPos10b = zeros(r,1);
count = 1;
for i = 1:rows
   if yNeg10toPos10a(i,1) > 0
      yNeg10toPos10b(count,1) = yNeg10toPos10a(i,1);
      count = count+1;
```

```
    end
end
% Finding Median and loading results
yNeg10toPos10 = median(yNeg10toPos10b(:,1));
xNeg10toPos10 = 0;

% Loading Median Area into Matrix
MedianEUArea (1,5) = yNeg10toPos10;

% Finding Median Area for Exchange Units 10 to 30
yPos10toPos30a = zeros(rows,1);
for i = 1:rows
    if CoGrph(i,8)>10 && CoGrph(i,8)<=30
        yPos10toPos30a(i,1) = CoGrph(i,4);
    end
end
% Counting the number of values
count = 0;
for i = 1:rows
    if yPos10toPos30a(i,1) > 0
        count = count +1;
    end
end
r = count;
% Creating final matrices and loading results
yPos10toPos30b = zeros(r,1);
count = 1;
for i = 1:rows
    if yPos10toPos30a(i,1) > 0
        yPos10toPos30b(count,1) = yPos10toPos30a(i,1);
        count = count+1;
    end
end
% Finding Median and loading results
yPos10toPos30 = median(yPos10toPos30b(:,1));
xPos10toPos30 = 20;

% Loading Median Area into Matrix
MedianEUArea (1,6) = yPos10toPos30;

% Finding Median Area for Exchange Units 30 to 50
yPos30toPos50a = zeros(rows,1);
for i = 1:rows
    if CoGrph(i,8)>30 && CoGrph(i,8)<50
        yPos30toPos50a(i,1) = CoGrph(i,4);
    end
end
% Counting the number of values
count = 0;
for i = 1:rows
    if yPos30toPos50a(i,1) > 0
        count = count +1;
    end
end
```

```
r = count;
% Creating final matrices and loading results
yPos30toPos50b = zeros(r,1);
count = 1;
for i = 1:rows
    if yPos30toPos50a(i,1) > 0
        yPos30toPos50b(count,1) = yPos30toPos50a(i,1);
        count = count+1;
    end
end
% Finding Median and loading results
yPos30toPos50 = median(yPos30toPos50b(:,1));
xPos30toPos50 = 40;

% Loading Median Area into Matrix
MedianEUArea (1,7) = yPos30toPos50;

% Finding Median Area for Exchange Units 50 (Group 5)
yMedianEUAreaPos50 = median(yGrp5);

% Loading Median Marea into Matrix
MedianEUArea (1,8) = yMedianEUAreaPos50;

% Saving Median Area Matrix
dlmwrite('MedianEUArea.txt',MedianEUArea,'Delimiter',';');

% Creating a Blank Matrix to Accept EU Area Data in the Order:
   % Column 1: Control Red Cells
   % Column 2: Group 1
   % Column 3: Neg50toNeg30b
   % Column 4: Neg30toNeg10b
   % Column 5: Neg10toPos10b
   % Column 6: Pos10toPos30b
   % Column 7: Pos30toPos50b
   % Column 8: Group 5
   % Column 9: Control Green Cells
% And Loading and Saving as AreaEUData.txt

AreaEUData = zeros(cMAX,9);

sz = size (yContRed);
l = sz(1,1);
AreaEUData(1:l,1) = yContRed(1:l,1);

sz = size (yGrp1);
l = sz(1,1);
AreaEUData(1:l,2) = yGrp1(1:l,1);

sz = size (yNeg50toNeg30b);
l = sz(1,1);
AreaEUData(1:l,3) = yNeg50toNeg30b(1:l,1);

sz = size (yNeg30toNeg10b);
l = sz(1,1);
```

```
AreaEUData(1:l,4) = yNeg30toNeg10b(1:l,1);

sz = size (yNeg10toPos10b);
l = sz(1,1);
AreaEUData(1:l,5) = yNeg10toPos10b(1:l,1);

sz = size (yPos10toPos30b);
l = sz(1,1);
AreaEUData(1:l,6) = yPos10toPos30b(1:l,1);

sz = size (yPos30toPos50b);
l = sz(1,1);
AreaEUData(1:l,7) = yPos30toPos50b(1:l,1);

sz = size (yGrp5);
l = sz(1,1);
AreaEUData(1:l,8) = yGrp5(1:l,1);

sz = size (yContGreen);
l = sz(1,1);
AreaEUData(1:l,9) = yContGreen(1:l,1);

% Saving Area EU Data Matrix
dlmwrite('AreaEUData.txt',AreaEUData,'Delimiter',';');

clear l sz

clear count i r yNeg50toNeg30a yNeg10toPos10b yNeg30toNeg10a
clear yNeg10toPos10a yPos10toPos30a yPos30toPos50a MedianEUArea
clear AreaEUData


% MAKING THE PLOT

% Plotting Control Cells
x = xCont;
y = yCont;
subplot(2,3,5);
ylim([0 yAr])
s=60;
scatter(x,y,s,'^','MarkerEdgeColor',[0 0 0]);
hold on;

% Defining marker size
s=10;

% Plotting Group 1 Cells
x = xGrp1;
y = yGrp1;
subplot(2,3,5);
ylim([0 yAr])
scatter(x,y,s,'MarkerFaceColor',[1 0 0], 'MarkerEdgeColor',[1 0 0]);
hold on;
```

```
% Plotting Group 2 Cells
x = xGrp2;
y = yGrp2;
subplot(2,3,5);
ylim([0 yAr])
scatter(x,y,s,'MarkerFaceColor',[0.95 0.4 0.1],...
    'MarkerEdgeColor',[0.95 0.4 0.1]);
hold on;

% Plotting Group 3 Cells
x = xGrp3;
y = yGrp3;
subplot(2,3,5);
ylim([0 yAr])
scatter(x,y,s,'MarkerFaceColor',[0.9292 0.694 0.125],...
    'MarkerEdgeColor',[0.9292 0.694 0.125]);
hold on;

% Plotting Group 4 Cells
x = xGrp4;
y = yGrp4;
subplot(2,3,5);
ylim([0 yAr])
scatter(x,y,s,'MarkerFaceColor',[0.2 0.9 0.1],...
    'MarkerEdgeColor',[0.2 0.9 0.1]);
hold on;

% Plotting Group 5 Cells
x = xGrp5;
y = yGrp5;
subplot(2,3,5);
ylim([0 yAr])
scatter(x,y,s,'MarkerFaceColor',[0 0.5 0],...
    'MarkerEdgeColor',[0 0.5 0]);
hold on;

% Defining Triangle Size for Median Plots of Controls
s = 250;

% Plotting Median Value Area Control Green
x = xMedGreenCont;
y = yMedGreenCont;
subplot(2,3,5);
ylim([0 yAr])
scatter(x,y,s,'^','MarkerFaceColor',[0 0.5 0],...
    'MarkerEdgeColor',[0 0 0]);
hold on;

% Plotting Median Value Area Control Red
x = xMedRedCont;
y = yMedRedCont;
subplot(2,3,5);
ylim([0 yAr])
scatter(x,y,s,'^','MarkerFaceColor',[1 0 0],...
```

```
    'MarkerEdgeColor',[0 0 0]);
hold on;

% Defining Square Size for Median Plots of Co-Cultures
s = 150;

% Plotting Median Value Area -50
x = -50;
y = yMedianEUAreaNeg50;
subplot(2,3,5);
scatter(x,y,s,'s','MarkerFaceColor',[0 0 0],...
    'MarkerEdgeColor',[0 0 0]);
hold on;

% Plotting Median Value Area -50 to -30
x = xNeg50toNeg30;
y = yNeg50toNeg30;
subplot(2,3,5);
ylim([0 yAr])
scatter(x,y,s,'s','MarkerFaceColor',[0 0 0],...
    'MarkerEdgeColor',[0 0 0]);
hold on;

% Plotting Median Value Area -30 to -10
x = xNeg30toNeg10;
y = yNeg30toNeg10;
subplot(2,3,5);
ylim([0 yAr])
scatter(x,y,s,'s','MarkerFaceColor',[0 0 0],...
    'MarkerEdgeColor',[0 0 0]);
hold on;

% Plotting Median Value Area -10 to 10
x = xNeg10toPos10;
y = yNeg10toPos10;
subplot(2,3,5);
ylim([0 yAr])
scatter(x,y,s,'s','MarkerFaceColor',[0 0 0],...
    'MarkerEdgeColor',[0 0 0]);
hold on;

% Plotting Median Value Area 10 to 30
x = xPos10toPos30;
y = yPos10toPos30;
subplot(2,3,5);
ylim([0 yAr])
scatter(x,y,s,'s','MarkerFaceColor',[0 0 0],...
    'MarkerEdgeColor',[0 0 0]);
hold on;

% Plotting Median Value Area 30 to 50
x = xPos30toPos50;
y = yPos30toPos50;
subplot(2,3,5);
```

```
ylim([0 yAr])
scatter(x,y,s,'s','MarkerFaceColor',[0 0 0],...
    'MarkerEdgeColor',[0 0 0]);
hold on;

% Plotting Median Value Area 50
x = 50;
y = yMedianEUAreaPos50;
subplot(2,3,5);
scatter(x,y,s,'s','MarkerFaceColor',[0 0 0],...
    'MarkerEdgeColor',[0 0 0]);
hold on;

% Labelling the scattergram
title('Area')
xlabel('Exchange Units')
ylabel('Area (Sq Micrometers)')
hold on;

clear s x xCoClt xCont xContGreen xContRed xGrp1 xGrp2 xGrp3
clear xGrp4 xGrp5 y yCont yContGreen yContRed yGrp1 yGrp1 yGrp2
clear yGrp3 yGrp4 yGrp5 xMedGreenCont xRedCont xNeg10toPos10
clear xNeg30toNeg10 xNeg50toNeg30 xPos10toPos30 xPos30toPos50
clear yMedGreenCont yNeg10toPos10 yNeg30toNeg10 yNeg30toNeg10b
clear yNeg50toNeg30 yNeg50toNeg30b yPos10toPos30 yPos10toPos30b
clear yPos30toPos50 yPos30toPos50b xMedRedCont yMedRedCont rows yAr
clear yMedianEUAreaNeg50 yMedianEUAreaPos50 cMAX MedianGroupArea

% END SCRIPT
```
